# SKI is critical to counter TGF-β signaling to promote T cell function and autoimmunity

**DOI:** 10.1126/sciadv.aeb7051

**Published:** 2026-08-19

**Authors:** Junying Wang, Ziyi Chen, Hongrui Li, Zengli Guo, Gang Wang, Junnian Zheng, Yisong Y. Wan

**Affiliations:** ^1^ Lineberger Comprehensive Cancer Center, University of North Carolina at Chapel Hill, Chapel Hill, NC 27599, USA.; ^2^ Department of Microbiology-Immunology, University of North Carolina at Chapel Hill, Chapel Hill, NC 27599, USA.; ^3^ Cancer Institute, Xuzhou Medical University, Xuzhou, Jiangsu 221004, China.

## Abstract

Transforming growth factor–β (TGF-β) is central to suppressing T cell function to maintain tolerance and immune homeostasis. The current TGF-β signaling paradigm is however inadequate in explaining how TGF-β controls T cell function. Here, we found a previously unappreciated, SKI-dependent mechanism of TGF-β signaling to better explain how TGF-β restricts T cell function to bolster tolerance. We found that SKI protein was up-regulated in activated T cells and down-regulated in response to TGF-β, indicating a reciprocal relationship between SKI and TGF-β in controlling T cell function. T cell–specific SKI deletion ameliorated spontaneous lethal autoimmunity due to T cell–specific TGFβRII deletion and mitigated MOG/CFA-induced experimental autoimmune encephalomyelitis. SKI was required for activation-induced T cell function and related molecular programs. Therefore, inhibition of TGFβR not only results in the loss of Smad-dependent function but, more importantly, leads to the gain of SKI-dependent activity, which is essential for T cell–mediated autoimmunity. A critical mechanism underlying TGF-β–mediated suppression of T cell function is overcoming SKI activity.

## INTRODUCTION

Transforming growth factor–β (TGF-β) is an evolutionarily conserved pleiotropic cytokine that plays multifaceted roles in regulating immune response in a context-dependent manner (*1*, *2*). A fundamental function of TGF-β is to promote immune tolerance and homeostasis. The perturbation of TGF-β signaling leads to various autoimmune and inflammatory manifestations (*3*–*5*). T cells are particularly important in TGF-β–mediated effect because deregulation of TGF-β signaling specifically in T cells causes similar conditions (*6*–*8*). In addition, TGF-β is instrumental in directing T helper 17 (T_H_17) cell differentiation in the presence of interleukin-6 (IL-6) and IL-21 to contribute to autoimmune and inflammatory pathologies (*9*, *10*). It is of critical importance to understand the molecular mechanisms of TGF-β function in T cells.

The current paradigm suggests TGF-β signals in the following manner. The binding of TGF-β to its receptor (TGFβR) activates receptor-associated Smads (R-Smad2 and R-Smad3). Activated R-Smad2 and R-Smad3 associate with Smad4 to control the expression of target genes of TGF-β signaling (*11*–*14*). This paradigm appears generally applicable in T cells, because R-Smad2 and R-Smad3 are indeed required for TGF-β signaling in T cells to uphold self-tolerance (*15*–*17*). Yet, the abrogation of Smad4 in T cells, despite disrupting certain TGF-β–mediated effects including regulatory T cell (T_reg_ cell) induction, does not lead to autoimmunity in mouse and human. It instead leads to decreased T cell function and increased tumor development (*18*–*20*). In addition, Smad4 deletion led to a TGF-β–independent T_H_17 differentiation (*21*–*23*). These observations suggest that the current paradigm of TGF-β signaling in T cells is incomplete and needs further elucidation. In addition, the mechanisms underlying the essential role of Smad4 in promoting T cell function remain to be defined. SKI, a protein that is readily degraded upon TGF-β stimulation, counters TGF-β signaling by associating with Smad4 to restrain T_H_17 cell differentiation (*21*, *22*). It suggests that SKI is a critical checkpoint that TGF-β must overcome to promote T_H_17 cell differentiation. However, it remains unknown whether SKI has a function beyond controlling T_H_17 cell differentiation and whether SKI is involved in the fundamental roles of TGF-β in controlling T cell function in immune homeostasis and self-tolerance.

In this study, we found that, upon activation in vitro, T cells showed elevated SKI protein levels. TGF-β signaling was critical to inhibit such SKI protein elevation. In agreement, SKI protein was increased in T cells isolated from *Cd4Cre;Tgfbr2^fl/fl^* mice that developed early onset lethal autoimmunity. These observations suggested that SKI up-regulation promotes T cell functions to contribute to autoimmunity including lethal autoimmune disease in *Cd4Cre;Tgfbr2^fl/fl^* mice. By generating a T cell–specific SKI knockout mouse model, we found that simultaneous SKI deletion in *Cd4Cre;Tgfbr2^fl/fl^* mice led to reduced lethality and decreased T cell function. In addition, T cell–specific SKI knockout mice showed reduced experimental autoimmune encephalomyelitis (EAE) development with reduced T cell function. Further investigation revealed that SKI was critical for T cell proliferation and for countering TGF-β–mediated effects. This study not only found an essential role for SKI to promote T cell function but also revealed that a critical mechanism of TGF-β–mediated suppression of T cell function of restricting autoimmunity and bolstering tolerance is to overcome SKI’s function.

## RESULTS

### SKI was up-regulated in activated T cells and down-regulated by TGF-β

**Fig. 1.**
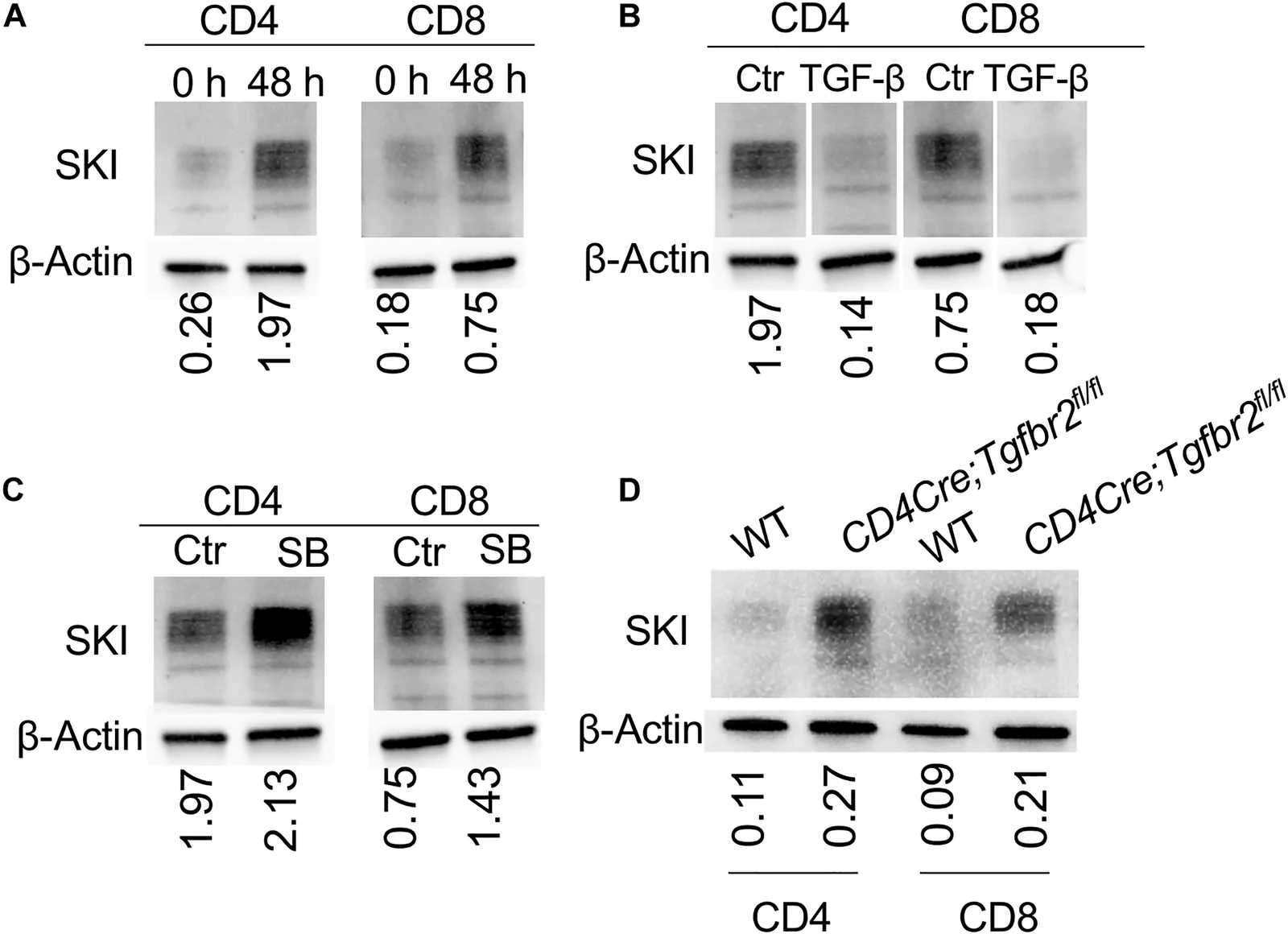
SKI protein levels in activated T cells with or without TGF-β signaling. (**A**) Immunoblotting of SKI protein in CD4^+^ and CD8^+^ T cells after being activated with plate-bound anti-CD3 (10 μg/ml) and anti-CD28 (5 μg/ml) for 48 hours. (**B**) Immunoblotting of SKI protein in CD4^+^ and CD8^+^ T cells after being activated with plate-bound anti-CD3 (10 μg/ml) and anti-CD28 (5 μg/ml) for 48 hours in the absence (Ctr) or presence of recombinant TGF-β (1 ng/ml). (**C**) Immunoblotting of SKI protein in CD4^+^ and CD8^+^ T cells after being activated with plate-bound anti-CD3 (10 μg/ml) and anti-CD28 (5 μg/ml) for 48 hours in the absence (Ctr) or presence of TGFβR inhibitor SB525334 (10 μM). Results of 48-hour–activated CD4 and CD8 T cells shown in (A) to (C) were from the same samples to allow for direct comparisons of different conditions. (**D**) Immunoblotting of SKI protein in CD4^+^ and CD8^+^ T cells isolated from the spleens and lymph nodes of 4-week-old wild-type (WT) and *Cd4Cre;Tgfbr2^fl/fl^* mice. The ratios between SKI and β-actin protein bands were determined by densitometry and indicated. Results are representative of three experiments.

As a pleiotropic cytokine, TGF-β controls diverse T cell functions. It inhibits T cell proliferation and T_H_1/T_H_2 cell differentiation (*24*, *25*), yet promotes T_H_17 cell differentiation (*9*, *10*). SKI has been shown to be a suppressor of T_H_17 cell differentiation by countering TGF-β signaling (*21*, *22*). Nonetheless, whether and how SKI functions beyond T_H_17 cell differentiation remains unaddressed. To address this question, we examined SKI protein expression during T cell activation. Both naïve CD4^+^ and CD8^+^ T cells had low levels of SKI protein. Yet, SKI protein levels were found greatly increased in CD4^+^ T cells after activation in the absence of T_H_ cell polarizing conditions (Fig. 1A and fig. S1A). Similarly, SKI protein was increased in activated CD8^+^ T cells (Fig. 1A and fig. S1A). Such SKI protein up-regulation became apparent at the time close to cell-cycle entry (24 to 48 hours postactivation) (*26*), indicating that it could be involved in regulating T cell proliferation. In addition, such SKI protein up-regulation can be regulated through multiple mechanisms including TCR, costimulation, and IL-2, because SKI protein up-regulation became less robust when CD28-mediated costimulation was absent and IL-2 was neutralized (fig. S1B). SKI up-regulation was exquisitely sensitive to TGF-β, because the addition of exogenous TGF-β greatly reduced SKI protein in activated CD4^+^ and CD8^+^ T cells (Fig. 1B). In addition, blocking endogenous TGF-β signaling with a TGFβR pharmacological inhibitor SB525334 further elevated SKI protein levels in activated CD4^+^ and CD8^+^ T cells (Fig. 1C). Similar observation was made under physiological settings. We found that, in a T cell–specific TGF-β receptor II knockout mouse strain *Cd4Cre;Tgfbr2^fl/fl^*, where T cells were spontaneously activated to cause an early lethal autoimmunity (*7*, *8*), SKI protein was substantially up-regulated in both CD4^+^ and CD8^+^ T cells isolated from these mice (Fig. 1D). These findings suggest that SKI is involved in fundamental functions of activated T cells beyond T_H_17 cell differentiation.

### SKI deletion mitigated lethal autoimmunity caused by TGFβR abrogation

The above-mentioned findings intrigued us to hypothesize that SKI up-regulation is important for the function of activated T cells and that one of the important mechanisms of TGF-β–mediated suppression of T cell function is to abrogate SKI function. To test this hypothesis, we first generated a T cell–specific *Cd4Cre;Ski^fl/fl^* mice, where exons 2 and 3 of *Ski* gene were specifically deleted in T cells (fig. S2A). SKI knockout in both CD4^+^ and CD8^+^ T cells was validated by immunoblotting (fig. S2B). *Cd4Cre;Ski^fl/fl^* mice were phenotypically grossly normal.

**Fig. 2.**
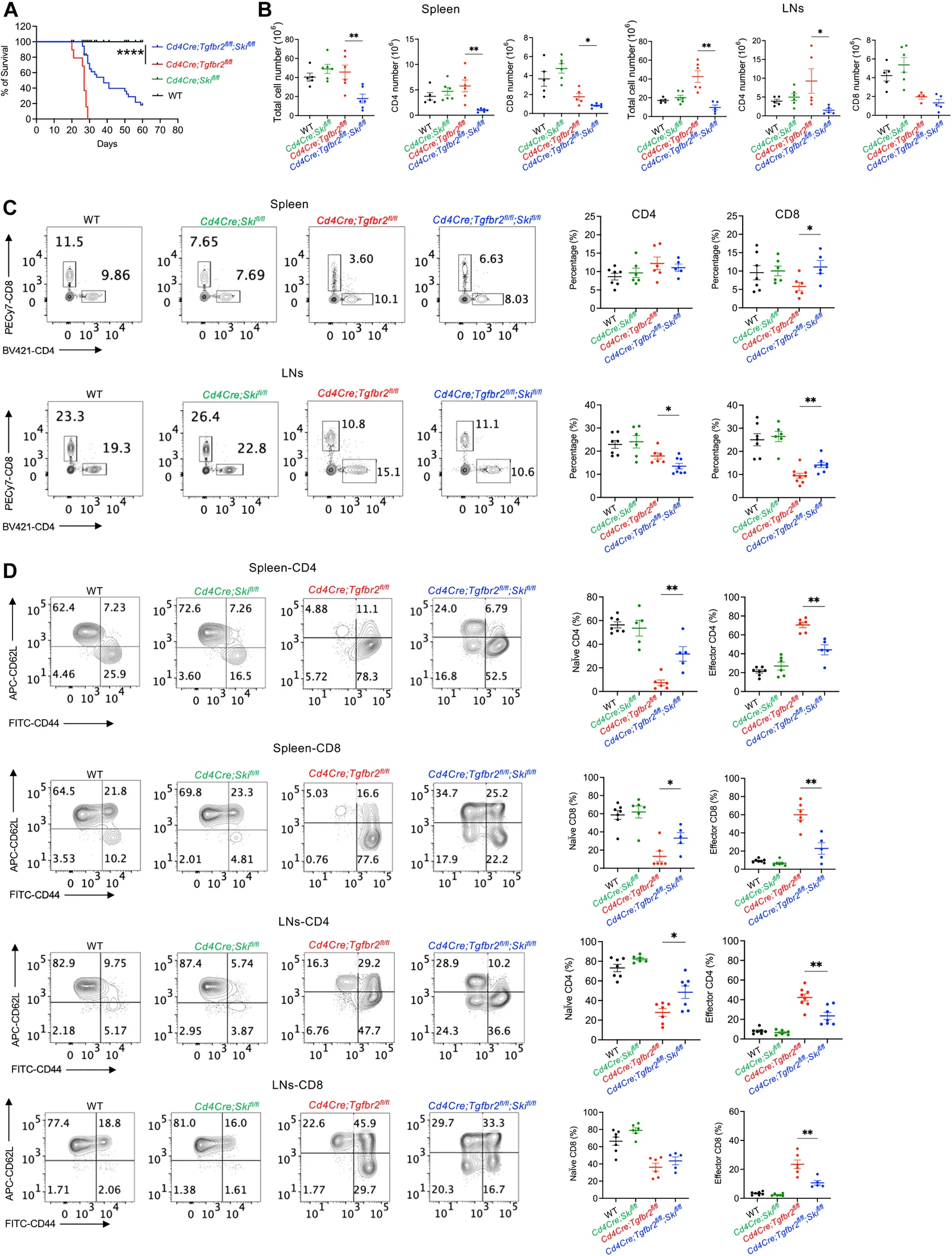
SKI deletion mitigated autoimmunity caused by TGFβRII abrogation in T cells. (**A**) The survival curve of WT, *Cd4Cre;Ski^fl/fl^*, *Cd4Cre;Tgfbr2^fl/fl^*, and *Cd4Cre;Tgfbr2^fl/fl^;Ski^fl/fl^* mice. *n* ≥ 19 for each group, ****P < 0.0001 per two-way analysis of variance (ANOVA). (**B**) The numbers of total lymphocytes, CD4^+^, and CD8^+^ T cells isolated from the spleens and peripheral lymph nodes of 3- to 4-week-old WT, *Cd4Cre;Ski^fl/fl^*, *Cd4Cre;Tgfbr2^fl/fl^*, and *Cd4Cre;Tgfbr2^fl/fl^;Ski^fl/fl^* mice. *n* ≥ 5 for each group, *P < 0.05; **P < 0.01 per *t* test. (**C**) Representative FACS plots showing CD4^+^ and CD8^+^ T cell percentages in the spleens and peripheral lymph nodes of WT, *Cd4Cre;Ski^fl/fl^*, *Cd4Cre;Tgfbr2^fl/fl^*, and *Cd4Cre;Tgfbr2^fl/fl^;Ski^fl/fl^* mice. *n* ≥ 5 for each group, *P < 0.05; **P < 0.01 per *t* test. (**D**) Surface expression of CD62L and CD44 on CD4^+^ and CD8^+^ T cells isolated from the spleens and peripheral lymph nodes of WT, *Cd4Cre;Ski^fl/fl^*, *Cd4Cre;Tgfbr2^fl/fl^*, and *Cd4Cre;Tgfbr2^fl/fl^;Ski^fl/fl^* mice. Naïve and effector T cells are categorized as CD62L^hi^CD44^lo^ and CD62L^lo^CD44^hi^, respectively. *n* ≥ 5 for each group, *P < 0.05; **P < 0.01 per *t* test.

To further investigate how SKI was involved in T cell–mediated spontaneous autoimmunity due to abrogation of TGF-β signaling, we crossed *Cd4Cre;Ski^fl/fl^* mice with *Cd4Cre;Tgfbr2^fl/+^* mice to obtain *Cd4Cre;Tgfbr2^fl/fl^;Ski^fl/fl^* mice to simultaneously delete TGFβRII and SKI in T cells. While *Cd4Cre;Tgfbr2^fl/fl^* mice succumbed to a multifocal autoimmune disease by 30 days after birth, *Cd4Cre;Tgfbr2^fl/fl^;Ski^fl/fl^* mice had a substantially extended life span (Fig. 2A). Therefore, SKI deletion significantly improved the survival of *Cd4Cre;Tgfbr2^fl/fl^* mice. To assess the cellular mechanisms underlying this observation, we analyzed and compared the populations and functions of T cells from mice of different genotypes. We found that *Cd4Cre;Ski^fl/fl^* mice had comparable total numbers of lymphocytes in the spleen and peripheral lymph nodes when compared to their wild-type (WT) littermates (Fig. 2B). CD4^+^ and CD8^+^ T cell numbers were similar between *Cd4Cre;Ski^fl/fl^* and WT mice (Fig. 2B). The distribution of CD4^+^ and CD8^+^ T cells in the spleens and peripheral lymph nodes was comparable between *Cd4Cre;Ski^fl/fl^* mice and their WT littermates (Fig. 2C). In addition, the distributions of naïve (CD62L^hi^CD44^lo^) and effector (CD62L^lo^CD44^hi^) T cells in the spleens and peripheral lymph nodes were similar between *Cd4Cre;Ski^fl/fl^* mice and their WT littermates (Fig. 2D). These findings suggest that SKI deletion did not perturb normal T cell generation and homeostasis.

Nonetheless, we found that the numbers of CD4^+^ and CD8^+^ T cells recovered from the spleens and lymph nodes in *Cd4Cre;Tgfbr2^fl/fl^;Ski^fl/fl^* mice were consistently lower when compared to *Cd4Cre;Tgfbr2^fl/fl^* mice (Fig. 2B). Further analysis revealed that the numbers of CD4^+^ T cells were consistently reduced in the spleens and lymph nodes in *Cd4Cre;Tgfbr2^fl/fl^;Ski^fl/fl^* mice when compared to *Cd4Cre;Tgfbr2^fl/fl^* mice (Fig. 2B). CD8^+^ T cell numbers were reduced in the spleen but not significantly changed in the periphery lymph nodes (Fig. 2B). The percentages of CD4^+^ and CD8^+^ T cells were similar between *Cd4Cre;Tgfbr2^fl/fl^;Ski^fl/fl^* and *Cd4Cre;Tgfbr2^fl/fl^* mice in spleen and lymph nodes (Fig. 2C). Compared with *Cd4Cre;Tgfbr2^fl/fl^* mice, *Cd4Cre;Tgfbr2^fl/fl^;Ski^fl/fl^* mice had higher proportions of naïve (CD62L^hi^CD44^lo^) CD4^+^ and CD8^+^ T cells in the spleen and lower proportions of effector (CD62L^lo^CD44^hi^) CD4^+^ and CD8^+^ T cells in both spleen and lymph nodes (Fig. 2D). *Cd4Cre;Tgfbr2^fl/fl^;Ski^fl/fl^* mice also showed an increase of pre–effector-like CD62L^−^CD44^−^ T cell populations (*27*) (fig. S3). It suggests that SKI deletion led to reduced T cell function when TGFβR signaling was abrogated in *Cd4Cre;Tgfbr2^fl/fl^;Ski^fl/fl^* mice.

**Fig. 3.**
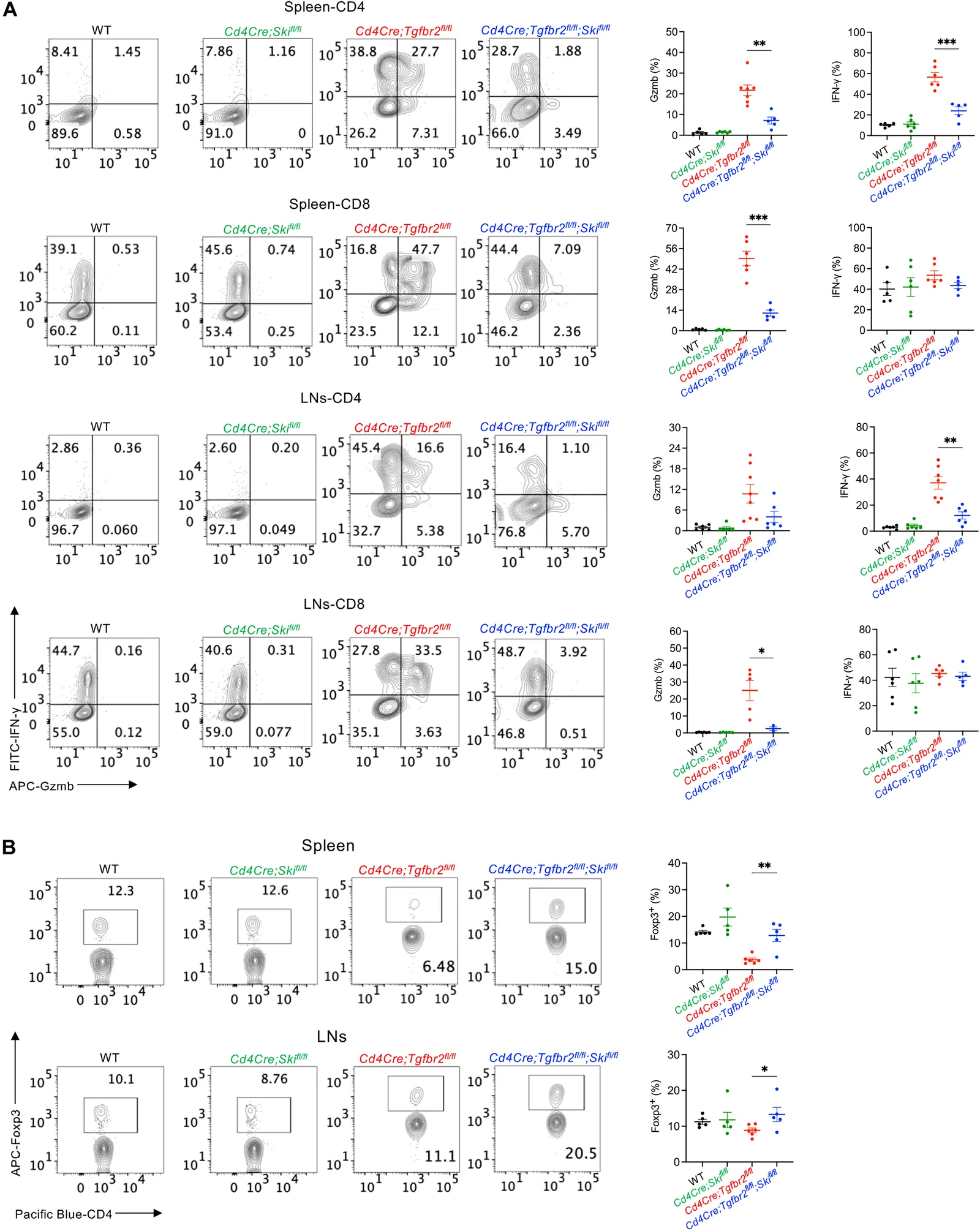
Cytokine production in T cells in WT, *Cd4Cre;Ski^fl/fl^*, *Cd4Cre;Tgfbr2^fl/fl^*, and *Cd4Cre;Tgfbr2^fl/fl^;Ski^fl/fl^* mice. (**A**) IFN-γ and GzmB production by CD4^+^ and CD8^+^ T cells isolated from the spleens and peripheral lymph nodes of WT, *Cd4Cre;Ski^fl/fl^*, *Cd4Cre;Tgfbr2^fl/fl^*, and *Cd4Cre;Tgfbr2^fl/fl^;Ski^fl/fl^* mice, assayed by intracellular staining and flow cytometry. *n* ≥ 5 for each group, *P < 0.05; **P < 0.01, ***P < 0.001 per *t* test. (**B**) Representative FACS plots showing Foxp3^+^ T cell percentages in CD4^+^ T cells in the spleens and peripheral lymph nodes of WT, *Cd4Cre;Ski^fl/fl^*, *Cd4Cre;Tgfbr2^fl/fl^*, and *Cd4Cre;Tgfbr2^fl/fl^;Ski^fl/fl^* mice. *n* ≥ 5 for each group, *P < 0.05; **P < 0.01 per *t* test.

To further analyze T cell effector function, we assessed the production of cytokine IFN-γ and effector molecule Granzyme B (Fig. 3A). T cells isolated from *Cd4Cre;Ski^fl/fl^* and WT mice produced comparable low amounts of IFN-γ and Granzyme B, in agreement with the observation that SKI deletion did not affect normal T cell homeostasis. As expected, Granzyme B production was markedly increased in CD4^+^ and CD8^+^ T cells when TGFβRII was abrogated in *Cd4Cre;Tgfbr2^fl/fl^* mice. Yet, simultaneous SKI deletion led to a substantial reduction of Granzyme B production in these T cells (Fig. 3A). Similarly, while IFN-γ production was markedly increased in CD4^+^ cells isolated from *Cd4Cre;Tgfbr2^fl/fl^* mice, such an increase was attenuated when SKI was also abrogated (Fig. 3A). In addition, we found that while the proportion of T_reg_ cells was decreased in *Cd4Cre;Tgfbr2^fl/fl^*, it was not apparently affected in *Cd4Cre;Tgfbr2^fl/fl^;Ski^fl/fl^* mice. Collectively, these findings suggest that SKI is critical for T cell function due to TGFβRII abrogation, to contribute to the reduced lethal autoimmunity observed in *Cd4Cre;Tgfbr2^fl/fl^;Ski^fl/fl^* mice.

### Alterations of *Cd4Cre;Tgfbr2^fl/fl^;Ski^fl/fl^* T cells were cell intrinsic

**Fig. 4.**
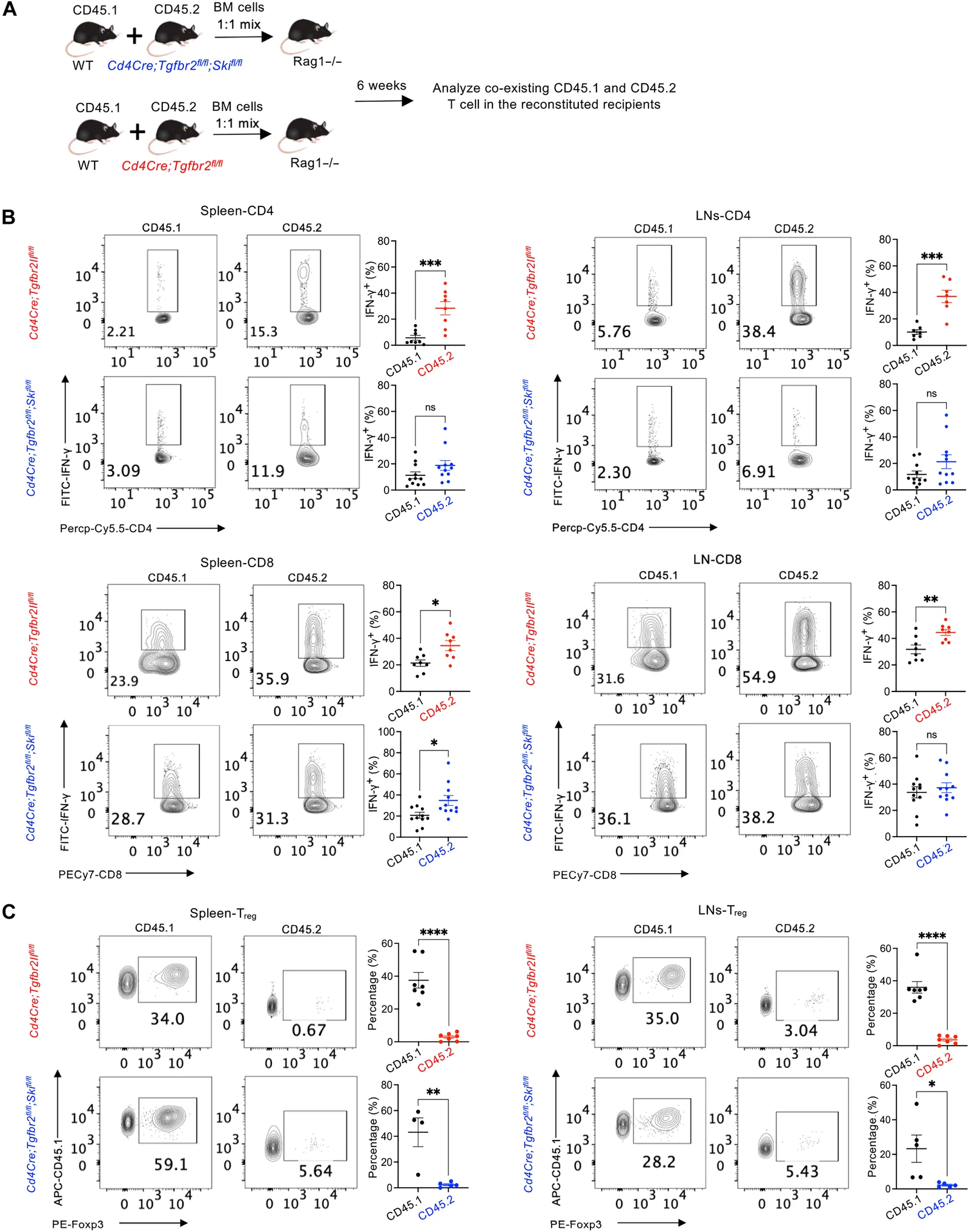
Cell-intrinsic changes of *Cd4Cre;Tgfbr2^fl/fl^;Ski^fl/fl^* T cells. (**A**) Schematic of generating mixed bone marrow chimera mice. Bone marrow cells isolated from *Cd4Cre;Tgfbr2^fl/fl^* (CD45.2) or *Cd4Cre;Tgfbr2^fl/fl^;Ski^fl/fl^* mice (CD45.2) were mixed at the ratio of 1:1 with the bone marrow cells from WT mice (CD45.1). Mixed bone marrow cells were then transferred into irradiated *Rag1^−/−^* recipients. Six weeks later, T cells were recovered from the recipient for analysis. (**B**) IFN-γ production by CD4^+^ and CD8^+^ T cells recovered from spleen and peripheral lymph nodes of the mixed bone marrow chimeric mice that contain *Cd4Cre;Tgfbr2^fl/fl^* (CD45.2) and WT (CD45.1) T cells or *Cd4Cre;Tgfbr2^fl/fl^;Ski^fl/fl^* (CD45.2) and WT (CD45.1) T cells, assessed by intracellular staining and flow cytometry. *n* ≥ 7 for each group, *P < 0.05; **P < 0.01; ***P < 0.001; ns, not significant; per *t* test. (**C**) The percentages of Foxp3^+^ T_reg_ cells in CD4^+^ T cells recovered from spleen and peripheral lymph nodes of the mixed bone marrow chimeric mice that contain *Cd4Cre;Tgfbr2^fl/fl^* (CD45.2) and WT (CD45.1) T cells or *Cd4Cre;Tgfbr2^fl/fl^;Ski^fl/fl^* (CD45.2) and WT (CD45.1) T cells, assessed by intracellular staining and flow cytometry. *n* ≥ 4 for each group, *P < 0.05; **P < 0.01; ****P < 0.0001; per *t* test.

To further investigate the cell-intrinsic effects of SKI deletion on TGFβRII-deficient T cells in vivo, we generated mixed bone marrow chimeric mice by transferring equal numbers of bone marrow cells from *Cd4Cre;Tgfbr2^fl/fl^;Ski^fl/fl^* mice (CD45.2) with WT mice (CD45.1), or *Cd4Cre;Tgfbr2^fl/fl^* mice (CD45.2) with WT mice (CD45.1) into irradiated *Rag1^−/−^* recipient mice (Fig. 4A). Compared to coexisting WT T cells in the fully reconstituted recipient mice, TGFβRII-deficient CD4^+^ and CD8^+^ T cells became activated with increased production of cytokine IFN-γ (Fig. 4B). In contrast, IFN-γ produced by SKI-TGFβRII–double-deficient T cells were largely comparable to that produced by coexisting WT mice in both spleens (Fig. 4B, left) and peripheral lymph nodes (Fig. 4B, right). Although the proportions of T_reg_ cells was found higher in *Cd4Cre;Tgfbr2^fl/fl^;Ski^fl/fl^* than in *Cd4Cre;Tgfbr2^fl/fl^* mice (Fig. 3B), *Cd4Cre;Tgfbr2^fl/fl^;Ski^fl/fl^* T_reg_ cells were greatly reduced, like *Cd4Cre;Tgfbr2^fl/fl^* T_reg_ cells, in the mixed bone marrow chimeras where WT T_reg_ cells coexisted (Fig. 4C). These results suggest that, although SKI deletion was unable to correct defective T_reg_ cell generation and homeostasis in the absence of TGFβRII, SKI deletion dampened conventional T cells’ function.

### SKI is essential for T cell–mediated neuroinflammatory EAE disease

**Fig. 5.**
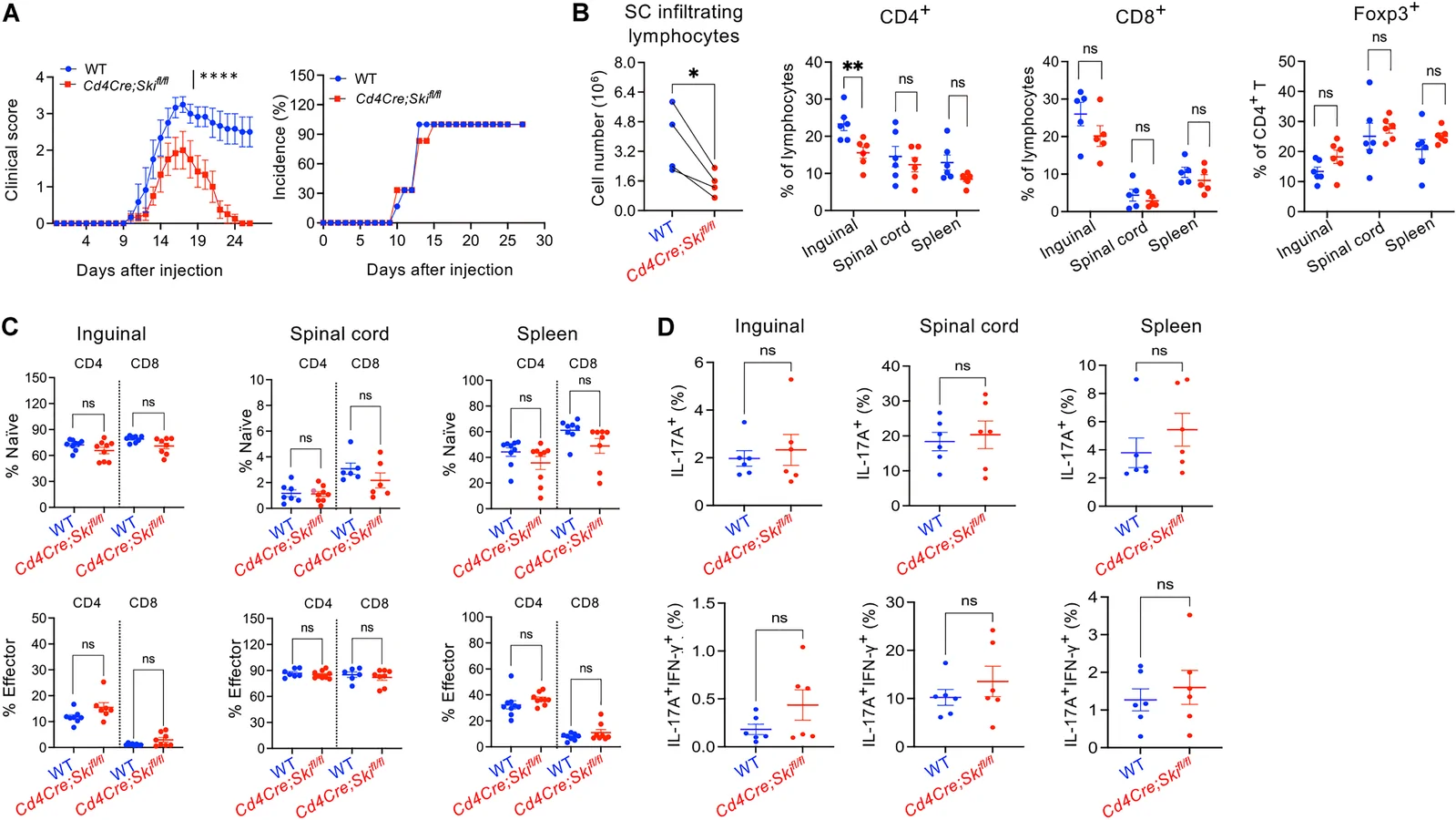
Ameliorated EAE development and reduced T cell expansion in *Cd4Cre;Ski^fl/fl^* mice. (**A**) The clinical scores and incidence of EAE disease induced by MOG/CFA in WT and *Cd4Cre;Ski^fl/fl^* mice. *n* = 6 for each group of two experiments, Means ± SEM. ****P < 0.0001 per two-way ANOVA. (**B**) The number of total lymphocytes infiltrated in the spinal cords at the peak of disease was counted. Each connected pair represents two mice matched for EAE clinical score at the time of analysis. *P < 0.05 per paired *t* test. The percentages of CD4^+^, CD8^+^, and T_reg_ cells in lymphocytes isolated from the draining inguinal lymph nodes, spinal cords, and spleens of EAE-inflicted WT and *Cd4Cre;Ski^fl/fl^* mice were determined by flow cytometry. *n* ≥ 4 for each group, **P < 0.01; ns, not significant per *t* test. (**C**) The percentages of naïve (CD62L^hi^CD44^lo^) and effector (CD62L^lo^CD44^hi^) CD4^+^ and CD8^+^ T cells were assessed by flow cytometry. *n* ≥ 6 for each group; ns, not significant per *t* test. (**D**) The percentages of IL-17A^+^T_H_17 and IL-17A^+^IFN-γ^+^ pathogenic T_H_17 cells in lymphocytes isolated from the draining inguinal lymph nodes, spinal cords, and spleens of EAE-inflicted WT and *Cd4Cre;Ski^fl/fl^* mice were determined by flow cytometry. *n* ≥ 6 for each group; ns, not significant per *t* test.

Intrigued by the finding that SKI was important for T cell–mediated spontaneous autoimmunity due to the abrogation of TGFβR, we further investigated whether SKI also contributes to T cell function in induced autoimmunity. To this end, we induced EAE disease, a mouse model that resembles human neuro-inflammatory autoimmune disease multiple sclerosis (*28*) in SKI-deficient *Cd4Cre;Ski^fl/fl^* mice and SKI-sufficient littermates (WT). Compared to SKI-sufficient littermates, *Cd4Cre;Ski^fl/fl^* mice developed less severe EAE, although EAE incidence showed no difference between these two groups (Fig. 5A). Notably, some of the EAE-inflicted *Cd4Cre;Ski^fl/fl^* mice experienced much improved recovery from the EAE disease (Fig. 5A). The reduced EAE development in *Cd4Cre;Ski^fl/fl^* mice might be due to compromised T cell expansion and/or function. To assess how T cell expansion and function are affected upon SKI deletion, we analyzed the numbers and cytokine production of spinal cord–infiltrating T cells. The numbers of spinal cord–infiltrating lymphocytes were lower in *Cd4Cre;Ski^fl/fl^* mice than in WT mice (Fig. 5B), agreeing with lower disease clinical scores in *Cd4Cre;Ski^fl/fl^* mice (Fig. 5A) and suggesting that SKI is required for the T cell expansion in the spinal cord during EAE. The percentages of spinal cord–infiltrating CD4^+^, CD8^+^, and T_reg_ cells were largely comparable between *Cd4Cre;Ski^fl/fl^* and WT mice (Fig. 5B), suggesting that SKI deletion did not preferentially affect the expansion of a particular T cell subset. The percentage of CD4^+^ T cells in *Cd4Cre;Ski^fl/fl^* mice was significantly reduced in the draining inguinal lymph nodes where T cells were primed for EAE development. We further analyzed the effector status of T cells isolated from inguinal, spinal cord, and spleen based on CD62L and CD44 surface expression. *Cd4Cre;Ski^fl/fl^* T cells became effectors normally (Fig. 5C). Similarly, *Cd4Cre;Ski^fl/fl^* T cells were found to produce IL-17A and IFN-γ normally (Fig. 5D). Collectively, these findings suggest that SKI deletion predominantly led to a defect in T cell expansion for a reduced EAE development in *Cd4Cre;Ski^fl/fl^* mice.

### Cell-intrinsic alterations of *Cd4Cre;Ski^fl/fl^* T cells during EAE development

**Fig. 6.**
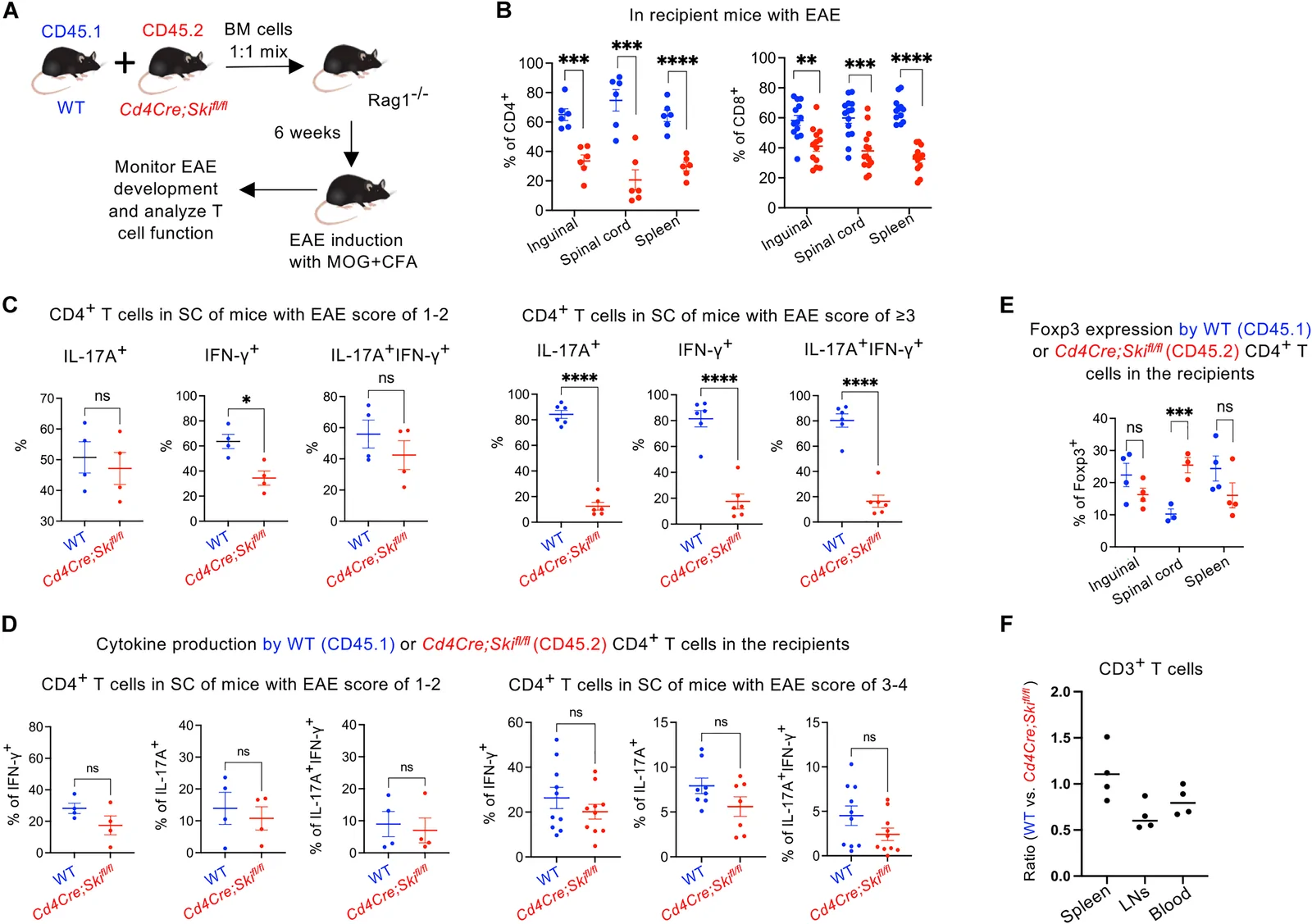
Cell-intrinsic defects of *Cd4Cre;Ski^fl/fl^* T cells during EAE. (**A**) Bone marrow cells isolated from *Cd4Cre;Ski^fl/fl^* mice (CD45.2) were mixed at the ratio of 1:1 with the bone marrow cells from WT mice (CD45.1). Mixed bone marrow cells were then transferred into irradiated *Rag1^−/−^* recipients. Six weeks later, EAE was induced in the recipients. (**B**) The proportions of coexisting WT (CD45.1, blue dots) and *Cd4Cre;Ski^fl/fl^* (CD45.2, red dots) in CD4^+^ and CD8^+^ T cells recovered from draining inguinal lymph nodes, spinal cords, and spleens in EAE-inflicted recipient mice, assessed by flow cytometry. *n* ≥ 6 for each group, **P < 0.01; ***P < 0.001; ****P < 0.0001; per *t* test. (**C**) The proportions of coexisting WT (CD45.1) and *Cd4Cre;Ski^fl/fl^* (CD45.2) CD4^+^ T cells that were IL-17A^+^, IFN-γ^+^, and IL-17A^+^IFN-γ^+^ in the spinal cords of the recipient mice with EAE clinical scores of 1 to 2 (EAE onset, left panel) and ≥3 (EAE peak, right panel), assessed by intracellular staining and flow cytometry. *n* ≥ 4 for each group, *P < 0.05; ****P < 0.0001; ns, not significant per *t* test. (**D**) The percentages of IL-17A^+^, IFN-γ^+^, and IL-17A^+^IFN-γ^+^ cells in WT and *Cd4Cre;Ski^fl/fl^* CD4^+^ T cells recovered from the spinal cord of EAE-inflicted recipient mice, assessed by intracellular staining and flow cytometry. *n* ≥ 4 for each group, ns, not significant per *t* test. (**E**) The percentages of Foxp3^+^ T_reg_ cells in WT (blue dots) and *Cd4Cre;Ski^fl/fl^* (red dots) CD4^+^ T cells recovered from the draining inguinal lymph nodes, spinal cords, and spleens of EAE-inflicted recipient mice, assessed by intracellular staining and flow cytometry. *n* ≥ 3 for each group, ***P < 0.001; ns, not significant per *t* test. (**F**) WT (CD45.1) and *Cd4Cre;Ski^fl/fl^* (CD45.2) bone marrow cells were mixed at a 1:1 ratio and transferred into irradiated recipient mice. The ratios between WT and *Cd4Cre;Ski^fl/fl^* CD3^+^ T cells in the spleen, LNs, and peripheral blood in the reconstituted recipient mice were determined by flow cytometry. Bars indicate the mean values.

Because the onset time and severity of EAE disease were different between *Cd4Cre;Ski^fl/fl^* mice and WT mice, it was challenging to identify a condition where *Cd4Cre;Ski^fl/fl^* mice and SKI-sufficient littermates displayed similar clinical scores at the same time (Fig. 5A). The differences of time points and/or disease scores chosen for analysis may exaggerate or mask some of the alterations of *Cd4Cre;Ski^fl/fl^* T cells. To unequivocally identify cell-intrinsic alterations of *Cd4Cre;Ski^fl/fl^* T cell function during EAE, we generated mixed bone marrow chimera mice where equal numbers of bone marrow cells from *Cd4Cre;Ski^fl/fl^* mice (CD45.2) and WT mice (CD45.1) were mixed and then transferred into irradiated *Rag1^−/−^* recipient mice. EAE was induced in fully reconstituted recipient mice (Fig. 6A). At different stages of EAE development, including onset (clinical score 1 to 2) and peak (clinical score 3 or above), lymphocytes were isolated from spleens, draining inguinal lymph nodes, and spinal cords of EAE-inflicted recipient mice. The composition and function of coexisting WT (CD45.1) and *Cd4Cre;Ski^fl/fl^* (CD45.2) T cells were assessed and compared. We found that the *Cd4Cre;Ski^fl/fl^* (CD45.2) T cells were outcompeted by coexisting WT (CD45.1) T cells in the spleen and draining inguinal lymph nodes and to a higher degree at inflammatory spinal cords, especially for CD4^+^ T cells (Fig. 6B). Similarly, IL-17A and IFN-γ cytokine-producing pathogenic T_H_ cells were dominated by WT (CD45.1) origin in the spinal cords, which become much more obvious at the peak of EAE (Fig. 6C). This WT dominance was not due to cell-intrinsic defects of cytokine production of *Cd4Cre;Ski^fl/fl^* T cells, because the percentages of IL-17A– and IFN-γ–producing cells among *Cd4Cre;Ski^fl/fl^* (CD45.2) CD4^+^ T cells were comparable to those of WT CD4^+^ T cells regardless of disease severity (Fig. 6D). Of note, the percentages of Foxp3^+^ T_reg_ cells among *Cd4Cre;Ski^fl/fl^* (CD45.2) CD4^+^ T cells in the spinal cords were higher than those of coexisting WT CD4 T^+^ cells (Fig. 6E), suggesting that SKI deletion has less impact on the expansion of T_reg_ cells than conventional T cells. The observed reduction of SKI KO T cells during EAE was mainly due to a defective response of SKI KO T cell to activation and inflammation, because without EAE induction, SKI KO T cell compartments are comparable to the coexisting WT T cell compartments in the mixed bone marrow chimeric mice (Fig. 6F). These findings suggest that SKI is required to promote activation-induced expansion of conventional T cells to contribute to autoimmunity in a cell-intrinsic manner.

### SKI deficiency led to decreased proliferation of activated T cells

**Fig. 7.**
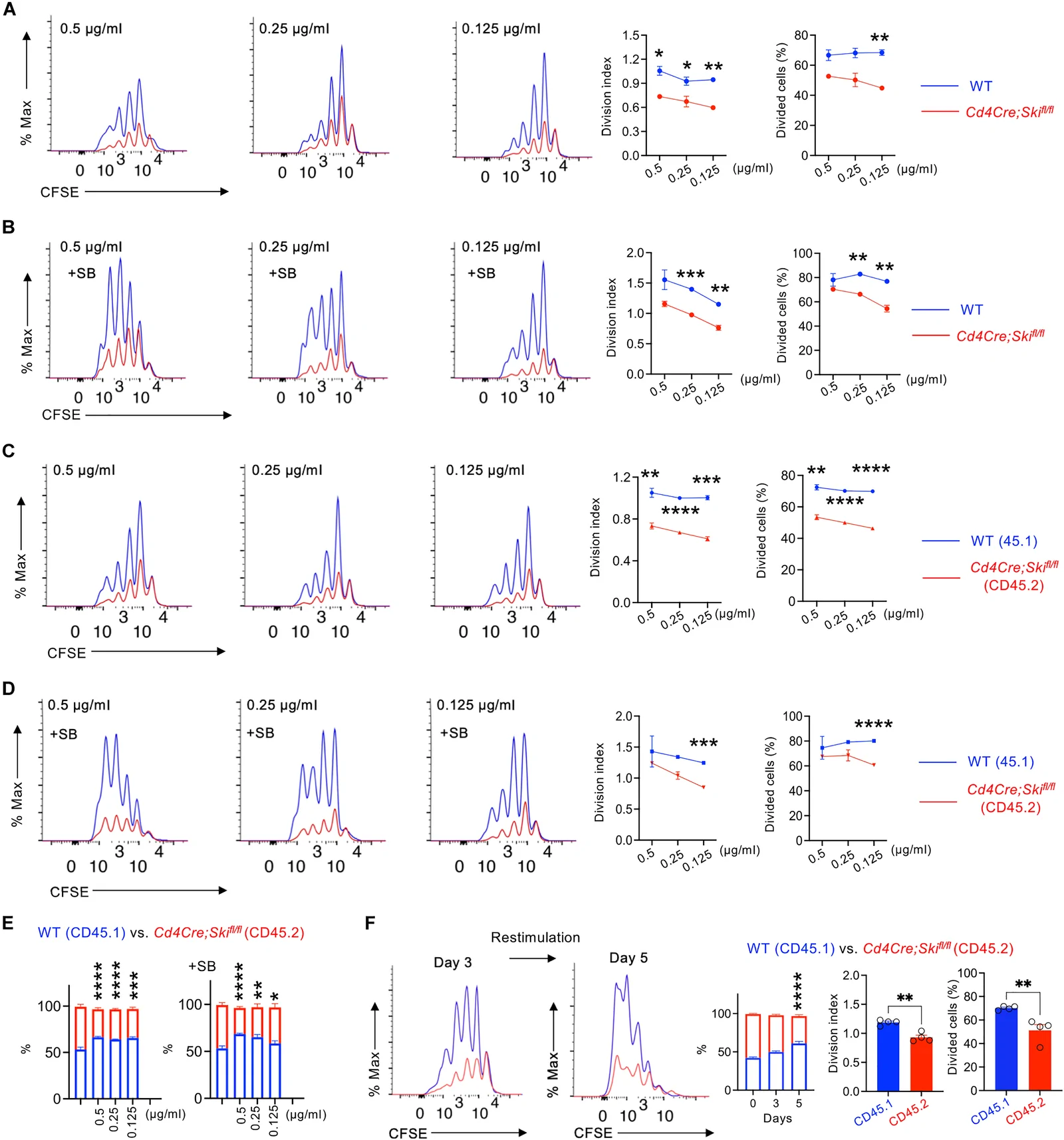
SKI deletion led to reduced proliferation of CD4^+^ T cells. (**A**) CFSE-labeled CD4^+^ T cells from WT and *Cd4Cre;Ski^fl/fl^* mice were activated by anti-CD3 at indicated concentrations with antigen-presenting cells for 3 days. Division index and percentages of divided cells were analyzed by flow cytometry and FlowJo software. (**B**) CFSE-labeled CD4^+^ T cells from WT and *Cd4Cre;Ski^fl/fl^* mice were activated by anti-CD3 at indicated concentrations with antigen-presenting cells for 3 days, in the presence of TGFβR inhibitor SB525334 (10 μM). Division index and percentages of divided cells were analyzed by flow cytometry and FlowJo software. (**C**) CFSE-labeled WT (CD45.1) and *Cd4Cre;Ski^fl/fl^* (CD45.2) CD4^+^ T cells were mixed at the ratio of 1:1. Cell mixtures were activated by anti-CD3 at indicated concentrations with antigen-presenting cells for 3 days. Division index and percentages of divided cells of coexisting WT (CD45.1) and *Cd4Cre;Ski^fl/fl^* (CD45.2) CD4^+^ T cells were analyzed by flow cytometry and FlowJo software. (**D**) CFSE-labeled WT (CD45.1) and *Cd4Cre;Ski^fl/fl^* (CD45.2) CD4^+^ T cells were mixed at the ratio of 1:1. Cell mixtures were activated by anti-CD3 at indicated concentrations with antigen-presenting cells for 3 days in the presence of TGFβR inhibitor SB525334 (10 μM). Division index and percentages of divided cells of coexisting WT (CD45.1) and *Cd4Cre;Ski^fl/fl^* (CD45.2) CD4^+^ T cells were analyzed by flow cytometry and FlowJo software. (**E**) The percentages of coexisting WT (CD45.1) and *Cd4Cre;Ski^fl/fl^* (CD45.2) CD4^+^ T cells at the end of the experiments as described in (C) and (D) were determined by flow cytometry. (**F**) Cell mixtures recovered from (C) were restimulated with plate-bound anti-CD3 for another 2 days. CFSE dilution, the percentages of coexisting WT (CD45.1) and *Cd4Cre;Ski^fl/fl^* (CD45.2) CD4^+^ T cells, division index, and percentage of divided cells were assessed by flow cytometry and FlowJo software. Means ± SEM, *n* ≥ 3. *P < 0.05, **P < 0.01, ***P < 0.001, ****P < 0.0001 per *t* test.

One of the most consistent observations in the models of spontaneous autoimmunity and induced autoimmune EAE was that SKI deletion led to substantially reduced CD4^+^ T cell expansion. This observation made us wonder whether the proliferation of T cells was affected by SKI deletion. To assess T cell proliferation capacity, we activated live-cell-tracer CFSE-labeled CD4^+^ T cells with different concentrations of anti-CD3 antibodies in the presence of antigen-presenting cells (APC). We found that, compared to SKI-sufficient CD4^+^ T cells, *Cd4Cre;Ski^fl/fl^* CD4^+^ T cells showed reduced proliferation (Fig. 7A). We also noticed that *Cd4Cre;Ski^fl/fl^* and WT T cells were activated similarly shortly after TCR stimulation, assessed by CD25 and CD69 up-regulation (figs. S4 and S5, A and B). It suggests that SKI is predominantly involved in T cell proliferation rather than immediate activation. These findings agree with the late SKI protein up-regulation (after 24 hours) postactivation (fig. S1). To investigate the impact of SKI deletion on T cell proliferation when TGF-β signaling was blocked, we added pharmacological TGFβR inhibitor SB525334 to the culture. We found that inhibition of TGFβR substantially enhanced the proliferation of SKI-sufficient CD4^+^ T cells per CFSE dilution. More importantly, such an enhancement was not observed in *Cd4Cre;Ski^fl/fl^* CD4^+^ T cells (Fig. 7B).

To further investigate whether the observed effects were cell intrinsic, we mixed equal numbers of WT (CD45.1) and *Cd4Cre;Ski^fl/fl^* (CD45.2) CD4^+^ T cells. The cell mixtures were labeled with CFSE and activated under the same conditions as described above. Consistent with the results of separated T cell culture, *Cd4Cre;Ski^fl/fl^* (CD45.2) CD4^+^ T cells proliferated less than coexisting WT CD4^+^ T cells (Fig. 7C) and remained unresponsive to TGFβR inhibition (Fig. 7D). Of note, SKI-deficient CD4^+^ T cells were consistently outcompeted by the coexisting WT CD4^+^ T cells (Fig. 7E) under the same culturing conditions. In addition, upon restimulation, SKI-deficient effector CD4^+^ T cells that were recovered 3 days after primary activation proliferated less when compared to WT counterparts under the same conditions (Fig. 7F). These observations agree with and contribute to the finding that, compared to coexisting SKI-sufficient counterparts, SKI-deficient CD4^+^ T cells in the spinal cords of EAE-inflicted mice became progressively less in number as disease progressed (Fig. 5B).

Like the observations made in CD4^+^ T cells, SKI-deficient CD8^+^ T cells also proliferated less and were less responsive to TGFβR inhibition, albeit to a lesser extent than SKI-deficient CD4^+^ T cells (fig. S5, B and C). These findings may explain the observation that there was less reduction of SKI-deficient CD8^+^ T cells than that of SKI-deficient CD4^+^ T cells under autoimmune disease settings (Figs. 3A, 5B, and 6B). Collectively, these results highlight an essential role for SKI to promote T cell proliferation, even when TGFβR was inhibited.

### SKI-dependent molecular programs for the function of activated T cells

**Fig. 8.**
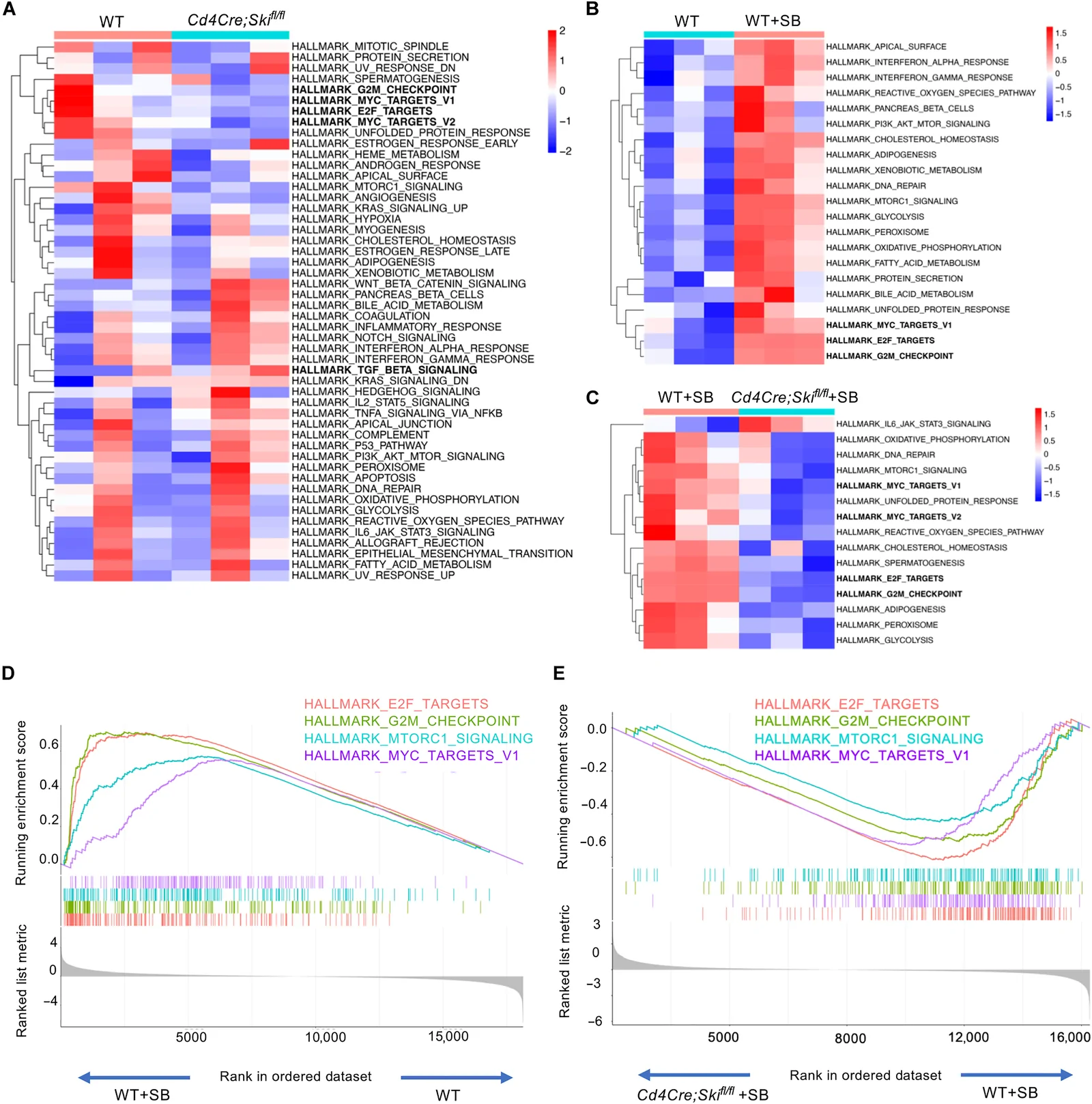
Molecular programs affected by SKI deletion in activated CD4 T cells. (**A**) GSVA comparing WT and *Cd4Cre;Ski^fl/fl^* CD4^+^ T cells after activation to reveal differences in the enrichment of hallmark gene sets. The pathways of interest are indicated in bold. Each column represents an individual biological replicate from one independent experiment of three (*n* = 3). Hallmark_G2M_CHECKPOINT: nominal *P* = 0.04, adjusted *P* = 0.74. HALLMARK_MYC_TARGETS_V1: nominal *P* = 0.06, adjusted *P* = 0.74. HALLMARK_E2F_TARGETS: nominal *P* = 0.09, adjusted *P* = 0.79. HALLMARK_MYC_TARGETS_V2: nominal *P* = 0.002, adjusted *P* = 0.12. (**B**) GSVA comparing WT CD4^+^ cells activated in the absence and presence (+) TGFβR inhibitor SB431542 (SB) to reveal differences in the enrichment of hallmark gene sets. The pathways of interest are indicated in bold. Each column represents an individual biological replicate from one independent experiment of three (*n* = 3). HALLMARK_MYC_TARGETS_V1: nominal *P* < 10^−6^, adjusted *P* < 10^−5^. HALLMARK_E2F_TARGETS: nominal *P* < 10^−8^, adjusted *P* < 10^−6^. Hallmark_G2M_CHECKPOINT: nominal *P* < 10^−6^, adjusted *P* < 10^−5^. (**C**) GSVA comparing WT and *Cd4Cre;Ski^fl/fl^* CD4^+^ T cells after activation in the presence of TGFβR inhibitor SB431542 (SB) to reveal differences in the enrichment of hallmark gene sets. The pathways of interest are indicated in bold. Each column represents an individual biological replicate from one independent experiment of three (*n* = 3). HALLMARK_MYC_TARGETS_V1: nominal *P* < 10^−3^, adjusted *P* < 10^−2^. HALLMARK_MYC_TARGETS_V2: nominal *P* < 10^−3^, adjusted *P* < 10^−2^. HALLMARK_E2F_TARGETS: nominal *P* < 10^−6^, adjusted *P* < 10^−3^. Hallmark_G2M_CHECKPOINT: nominal *P* < 10^−5^, adjusted *P* < 10^−2^. (**D**) GSEA to compare WT CD4^+^ T cells activated with or without SB treatment to show enhanced E2F, G2M, MTOR1, and Myc-target pathways in SB-treated samples. HALLMARK_E2F_TARGETS: nominal *P* = 0.003, adjusted *P* = 0.02, NES = 2.78. Hallmark_G2M_CHECKPOINT: nominal *P* = 0.003, adjusted *P* = 0.02, NES = 2.77. Hallmark_MTORC1_SIGNALING: nominal *P* = 0.003, adjusted *P* = 0.02, NES = 2.28. HALLMARK_MYC_TARGETS_V1: nominal *P* = 0.003, adjusted *P* = 0.02, NES = 2.21. (**E**) GSEA to compare WT and *Cd4Cre;Ski^fl/fl^* CD4^+^ T cells activated in the presence of SB to show reduced E2F, G2M, MTORC1, and Myc-target pathways in *Cd4Cre;Ski^fl/fl^* cells. HALLMARK_E2F_TARGETS: nominal *P* = 0.002, adjusted *P* = 0.04, NES = −2.49. Hallmark_G2M_CHECKPOINT: nominal *P* = 0.002, adjusted *P* = 0.04, NES = −2.42. Hallmark_MTORC1_SIGNALING: nominal *P* = 0.002, adjusted *P* = 0.04, NES = −1.89. HALLMARK_MYC_TARGETS_V1: nominal *P* = 0.002, adjusted *P* = 0.04, NES = −2.22.

To elucidate the molecular mechanisms underlying SKI-controlled T cell function, we mixed equal number of WT (CD45.1) and *Cd4Cre;Ski^fl/fl^* (CD45.2) CD4^+^ T cells and then activated the cell mixtures with anti-CD3 in the presence of APCs with or without TGFβR inhibitor SB525334. Three days after activation, WT (CD45.1) and *Cd4Cre;Ski^fl/fl^* (CD45.2) CD4^+^ T cells were sorted by fluorescence-activated cell sorting (FACS) and subjected to RNA sequencing (RNA-seq) analysis to assess what molecular programs were affected by SKI deletion in activated CD4^+^ T cells in a cell-intrinsic manner with or without TGF-β signaling. Consistent with the proliferation defect observed in *Cd4Cre;Ski^fl/fl^* T cells (Fig. 7A), the Myc-target and G2M-checkpoint gene sets, which are two canonical Hallmark programs associated with cell cycle progression and proliferation, trended lower in *Cd4Cre;Ski^fl/fl^* CD4^+^ T cells (Fig. 8A). Pathways related to TGF-β signaling showed directional enrichment (NES = 1.50) in *Cd4Cre;Ski^fl/fl^* cells, which agree with SKI’s function of countering TGF-β signaling (fig. S6). We further assessed what molecular programs are targeted by endogenous TGF-β to restrain T cell proliferation, because we observed a greatly enhanced T cell proliferation when endogenous TGF-β signaling was blocked by SB525334 in WT T cells (Fig. 7, B and D). Pathways related to Myc-target, E2F-target, and G2M-checkpoint were significantly up-regulated when TGF-β signaling was inhibited (Fig. 8B). Many SKI-dependent pathways, including Myc-target and G2M-checkpoint, were inversely affected by TGFβR inhibition (Fig. 8, B and C). Furthermore, we found that TGFβR inhibition failed to reverse the molecular changes due to SKI deletion, in particular the decreased expression of genes related to Myc-targets, E2F-target, and G2M-checkpoint pathways (Fig. 8C). Further gene set enrichment analysis (GSEA) confirmed that while TGFβR inhibition enhanced the expression of Myc-target, E2F-target, and G2M-checkpoint genes in WT cells, it did not restore these gene’s expression when SKI was deleted (Fig. 8, D and E). These results suggested that SKI is required for activation-induced molecular program changes for T cells to proliferate even when TGFβR was inhibited.

## DISCUSSION

The critical role for TGF-β in controlling diverse T cell functions is unequivocal. Nonetheless, the mechanisms of how TGF-β signaling pathways mediate these functions remain unclear and perplexing. According to the current paradigm, TGF-β predominantly signals through an rSmad-Smad4–dependent pathway (*11*–*14*). The current model is indeed useful in explaining some of the experimental observations made in T cells as rSmad2 and rSmad3 are required for TGF-β to suppress T_H_1 cell function and for TGF-β–induced T_reg_ cell generation (*15*–*17*). Nonetheless, when it comes to the function of Smad4 in T cells, the experimental observations are perplexing and not as predicted. Because Smad4 is the “common” Smad to mediate TGF-β signaling, it was predicted that the effects of deleting Smad4 in T cells would be like those of deleting TGFβR in T cells to develop autoimmunity (*6*–*8*). Quite to the contrary, it was found that mice with T cell–specific Smad4 deletion are phenotypically normal (*18*–*20*), and these mice even showed reduced ability to eradicate tumors (*18*, *29*), agreeing with the findings in human that familial Smad4 mutations often associate with high incidence of cancer but not autoimmunity (*30*, *31*). More intriguingly, simultaneous deletion of Smad4 and TGFβRII corrected the early lethal autoimmunity of T cell–specific TGFβRII knockout mice (*18*). Smad4 was found to be paradoxically required for the proliferation of activated T cells, although Smad4 remains required for TGFβ-induced T_reg_ cell generation (*18*). In addition, the current paradigm would predict that Smad4 should be required for TGF-β–induced T_H_17 cell generation. However, the opposite was observed. Smad4-deficient T cells can differentiate into T_H_17 cells to promote EAE development, even when TGF-β signaling was abrogated (*21*, *22*). It is now realized that, when TGF-β signal was low or absent, Smad4 associates with SKI protein to counteract TGF-β to restrict the transactivation of T_H_17-related loci that bind constitutively by Smad4. SKI and TGF-β function in an antagonistic manner. SKI competes with rSmads for Smad4 binding (*32*) and negatively regulates certain TGF-β responsive genes (*33*). Conversely, TGF-β triggers SKI protein degradation (*34*, *35*). TGFβ-induced SKI degradation is critical for T_H_17 differentiation, as ectopic SKI expression hampered T_H_17 cell differentiation and EAE development (*21*, *22*, *36*). These findings suggest that, for T_H_17 cell differentiation, SKI is a critical “checkpoint” that TGFβ signaling must overcome for its effects to promote T_H_17 cell differentiation. In the current study, we revealed that SKI protein was up-regulated in T cells upon activation and down-regulated by TGF-β, suggesting that SKI may counter TGF-β to promote T cell function and autoimmunity. SKI deletion mitigated T cell–mediated early lethal autoimmunity due to TGFβR abrogation and was required for MOG/CFA-induced, T cell–mediated EAE disease. SKI was required for activation-induced T cell expansion and proliferation. In addition, in the absence of TGFβR, SKI deletion also led to an increase of CD62L^−^CD44^−^ T cell populations proposed to be pre–effector-like (*27*) to correlate and potentially contribute to reduced effector T cell function and immune pathology in these mice, which warrants further investigation. These results collectively support a revised model to fully appreciate how TGF-β controls T cell function and autoimmunity. SKI, a largely ignored critical component of TGF-β signaling, needs to be integrated as the following: By default, SKI dependent checkpoint actively restrains TGFβR-rSmad dependent processes. Yet, TGFβR activation leads to SKI degradation and thus the disruption of SKI-dependent checkpoints to allow the engagement of TGFβR-rSmad dependent processes. Both SKI-dependent and r-Smad–dependent pathways are active processes critical for T cell function and reciprocally controlled by TGF-β. Interfering with TGF-β signaling will not merely disrupt Smad-dependent function but also activate SKI-dependent function that is essential for T cell function. Much investigation is therefore warranted to reveal the genetic, epigenetic, and molecular mechanisms underlying SKI-dependent checkpoint pathway and how it functionally interacts with rSmad-dependent pathways in controlling T cell function and autoimmunity.

We found that simultaneous SKI deletion corrected the early lethal autoimmunity due to TGFβR deletion in T cells. Nonetheless, such a correction is not as complete as when Smad4 was simultaneously deleted with TGFβRII in T cells (*18*). These findings suggest that additional TGF-β/Smad4–dependent factor(s) may also contribute to the checkpoint function. Our previous study found that besides SKI, Smad4 also associates with SnoN in T cells (*21*). SnoN is a protein closely related to SKI and has similar function and regulation as SKI does (*37*). Like SKI, SnoN is up-regulated in activated T cells and down-regulated by TGF-β (*38*). It was found that SnoN deletion led to defective T cell activation and augmented TGF-β signaling (*39*). It is therefore plausible that SKI coordinated with SnoN to actively control the checkpoint of TGF-β signaling in T cells. It would be of interest to investigate how SKI and SnoN may function redundantly and/or synergistically to counter TGF-β signaling in controlling T cell function and autoimmunity.

TGF-β broadly controls the functions of diverse immune cell types including T cells, NK cells, and ILCs. Paradoxical observations that Smad4 plays an important role to promote the functions of these cells were made under various conditions (*20*, *40*–*43*). However, the molecular mechanisms underlying these observations remain to be elucidated. We found that SKI-deficient T cells, like Smad4-deficient T cells, showed reduced T cell expansion and function to highlight SKI as a critical checkpoint that TGF-β needs to overcome to restrict T cell function and autoimmunity. It would be of interest to address whether this SKI-dependent checkpoint is also broadly involved in controlling the function of NK cells and ILCs in a TGF-β–dependent and –independent manner.

## MATERIALS AND METHODS

### Animals

*Cd4Cre;Tgfbr2^fl/fl^*, *Cd4Cre;Ski^fl/fl^*, *Cd4Cre;Tgfbr2^fl/fl^SKI^fl/fl^*, *Rag1^−/−^*, CD45.2, and CD45.1 mice were on C57BL/6 background. All mice were housed and bred under specific pathogen–free conditions in the animal facility at the University of North Carolina at Chapel Hill. All mouse experiments were performed using both male and female, age- and sex-matched mice (littermates when possible), and were approved by the Animal Care and Use Committee of the University of North Carolina at Chapel Hill (protocol no. 25-112).

### Lymphocyte isolation, T cell culture, and proliferation in vitro

Lymphocytes were collected from pooled lymph nodes and spleens. CD4^+^ and CD8^+^ T cells were purified by microbeads (CD4 L3T4, CD8 Ly-2, Miltenyi Biotec). T cells were activated by soluble anti-CD3 (145-2c11, BioXCell) in the presence of APCs unless stated otherwise. To assess proliferation, cells were labeled with 5 μM CFSE before activation. Cells were cultured in RPMI 1640 with 10% fetal bovine serum, 10 mM l-glutamine, 1% pen-strep antibiotics, and 60 μM β-mercaptoethanol. TGF-β receptor I (ALK5) inhibitor SB525334 was used to block TGF-β signaling pathway.

### Flow cytometry and cell sorting

Fluorescence-conjugated anti-CD4 (RM4–5), anti-CD8 (Ly-2), anti-CD45.1 (A20), anti-CD62L (MEL-14), anti-CD44 (IM7), anti-GZMB (QA16A02), anti–IFN-γ (XMG 1.2), anti–IL-17A (TC11-18H10.1), and anti-CD45.2 (104) were purchased from BioLegend, and anti-Foxp3 (FJK-16 s) was purchased from eBioscience. A total of 1 × 10^6^ cells were used for FACS staining. Fixation and permeabilization for intranuclear staining were performed according to the manufacturer’s protocols. For cytokine intracellular staining, cells were stimulated for 4 hours in medium with PMA (20 ng/ml; phorbol 12-myristate 13-acetate) and 1 μM ionomycin and brefeldin A (5 μg/ml). Cell samples were analyzed on BDSymphony A6 or a BDFortessa flow cytometer. For cell sorting, mixed cell culture was stained with anti-CD4, anti-CD8, anti-CD45.1, anti-CD45.2, CFSE, and live dye-APC-Cy7 (Zombie NIR, BioLegend). Stained cells were washed and sorted on BDFACS Aria II by the flow core facility at the University of North Carolina at Chapel Hill.

### RNA-seq and analysis

The sorted cells were lysed in Trisure reagent. Total RNA was extracted using the Zymo RNA microprep kit per the manufacturer’s instruction. RNA-seq libraries were generated and sequenced as previously described (*44*, *45*). The raw reads were filtered by removing adaptor sequences, contamination, and low-quality reads. Cleaned reads were aligned to the mm10 reference genome using “Hisat2” (Version 2.2.1). The quantification results from “featureCount (subread, Version 2.1.1)” were then analyzed with the R packages “DESeq2,” “edgeR,” and “limma” to identify common differentially expressed genes [DEGs, adjusted *P* value <0.05 and log_2_(fold change) > 1.5]. The R packages “clusterProfiler” (version 4.17.0) and “GSVA” (version 2.2.0) were used to perform GSEA and gene set variation analysis (GSVA). Gene sets in the Molecular Signatures Database were selected as the reference gene sets, and an adjusted *P* value ≤0.25 per GSEA and adjusted *P* value ≤0.05 per GSVA were set as the threshold. R version 4.4.0 was used.

### Bone marrow chimera and EAE

Bone marrow cells were collected from age- and sex-matched WT C57BL/6 (CD45.1) and *Cd4Cre;Ski^fl/fl^*, *Cd4Cre;Tgfbr2^fl/fl^*, or *Cd4Cre;Tgfbr2^fl/fl^;Ski^fl/fl^* (CD45.2) mice, mixed at 1:1 ratio. A mixture of one million cells was transferred retro-orbitally into sublethal irradiated (4 Gy) *Rag1^−/−^* mice. After 6 weeks, recipients were subjected to analysis or EAE induction. For EAE induction, 25 mg of MOG_35–55_ peptide (MEVGWYRSPFSRVVHLYRNGK, AnaSpec) and 5 mg of *Mycobacterium tuberculosis* (Difco) were emulsified in IFA (Difco) and then injected subcutaneously into mice followed by intraperitoneal injection of 200 ng of Pertussis Toxin (List Biological Laboratories) on day 0 and day 2. The severity of EAE was monitored and graded on a clinical score of 1 to 6 by the following standard: 1, limp tail; 2, poor righting ability and/or partial hindlimb paralysis; 3, total hindlimb paralysis; 4, hindlimb paralysis +75% of body paralysis; 5, moribund; 6, dead. For spinal cord dissection, mice were perfused with ice-cold phosphate-buffered saline containing heparin (20 U/ml). The isolated spinal cords were cut into around 1-mm^3^ cubes and digested with collagenase D (1 mg/ml; Sigma-Aldrich) for 45 min at 37°C. The digested tissues were washed with 38% Percoll (Sigma-Aldrich) and centrifuged at 2000 rpm for 20 min at brake 1 to separate lymphocytes.

### Western blot

Activated CD4^+^ or CD8^+^ T cells were collected after 48 hours of culturing. Approximately 500,000 T cells were lysed in radioimmunoprecipitation assay buffer with protease inhibitor cocktail (Roche Molecular Biochemicals) on ice for 30 min and further mixed with 2× Laemmli sample buffer (Bio-Rad, 1610737) at 95°C for 10 min. The boiled sample was centrifuged at 15,000 rpm for 5 min and proteins were collected from the supernatant. Protein mixtures were separated on SDS–polyacrylamide gel electrophoresis gel at 100 V for 3 hours and transferred onto a preactivated polyvinylidene fluoride blot under 200 mA for 1.5 hours. The blot was blocked by 5% bovine serum albumin in TBST at room temperature for 1 hour and incubated with primary antibodies including anti–β-actin HRP (Santa Cruz, sc-4778, 1:5000) and anti-SKI (G8, Santa Cruz, 1:2000). After washing three times with TBST, secondary antibody donkey anti-mouse HRP (Jackson ImmunoResearch Laboratories, 715-035-150) was applied and incubated with blots at room temperature for 1 hour, followed by three times TBST washing. Protein bands were developed using ECL substrate solution (Thermo Fisher Scientific).

### Data analysis

Statistics was processed and presented by Prism (GraphPad, San Diego). Two-tailed Student’s *t* test was used to compare two groups of samples unless stated otherwise. A *P* value of less than 0.05 was considered significant.
