# Supplementary material for: SKI is critical to counter TGF-β signaling to promote T cell function and autoimmunity
Source: Sci Adv. 2026 Aug 19;12(34):eaeb7051. doi: 10.1126/sciadv.aeb7051 (PMC13488881; doi:10.1126/sciadv.aeb7051)
Supplement: Supplementary file 1 [file sciadv.aeb7051_sm.pdf]

Science Advances
AAAS

Supplementary Materials for

SKI is critical to counter TGF-β signaling to promote T cell function and autoimmunity

Junying Wang *et al.*

Corresponding author: Yisong Y. Wan, wany@email.unc.edu

Sci. Adv. **12**, eaeb7051 (2026)
DOI: 10.1126/sciadv.aeb7051

This PDF file includes:

Figs. S1 to S6

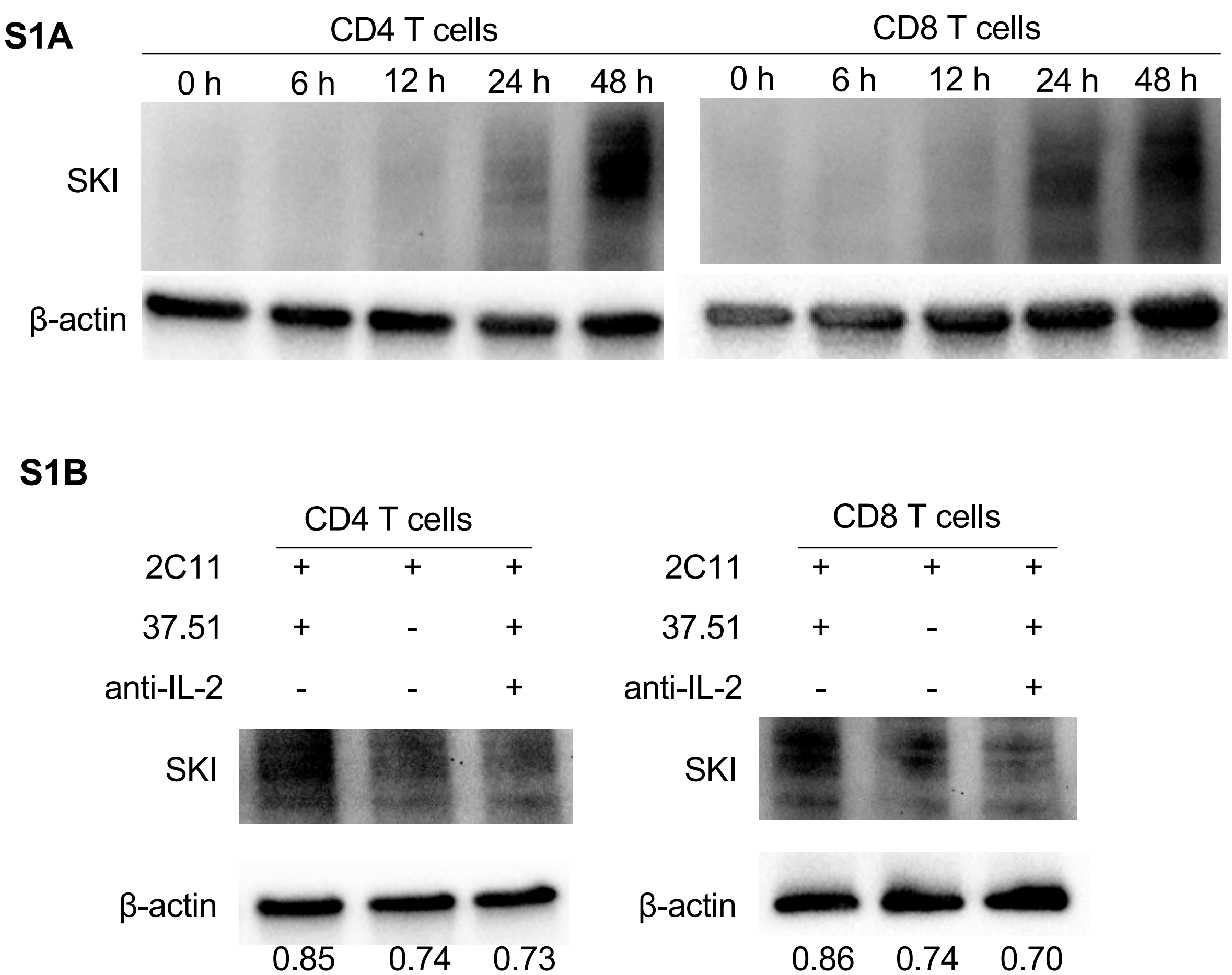

Supplementary Figure S1: SKI protein upregulation in activated T cells. 1A. Immunoblotting of SKI protein in $CD4^+$ and $CD8^+$ T cells after being activated with plate-bound anti-CD3 (10μg/ml) and anti-CD28 (5μg/ml) for 0, 6, 12, 24 and 48 hours (h). Results are representative of three experiments. **1B.** Immunoblotting of SKI expression in CD4+ and CD8+ T cells after being activated with plate bound anti-CD3 (1C11, 10 ug/ml), with (+) or without (-) anti-CD28 (37.51, 5 ug/ml), in the presence (+) or absence (-) of anti-IL-2 neutralizing antibody (10 ug/ml). The ratio between SKI to β-actin protein bands were determined by densitometry and indicated. Results are representative of three experiments.

S2A

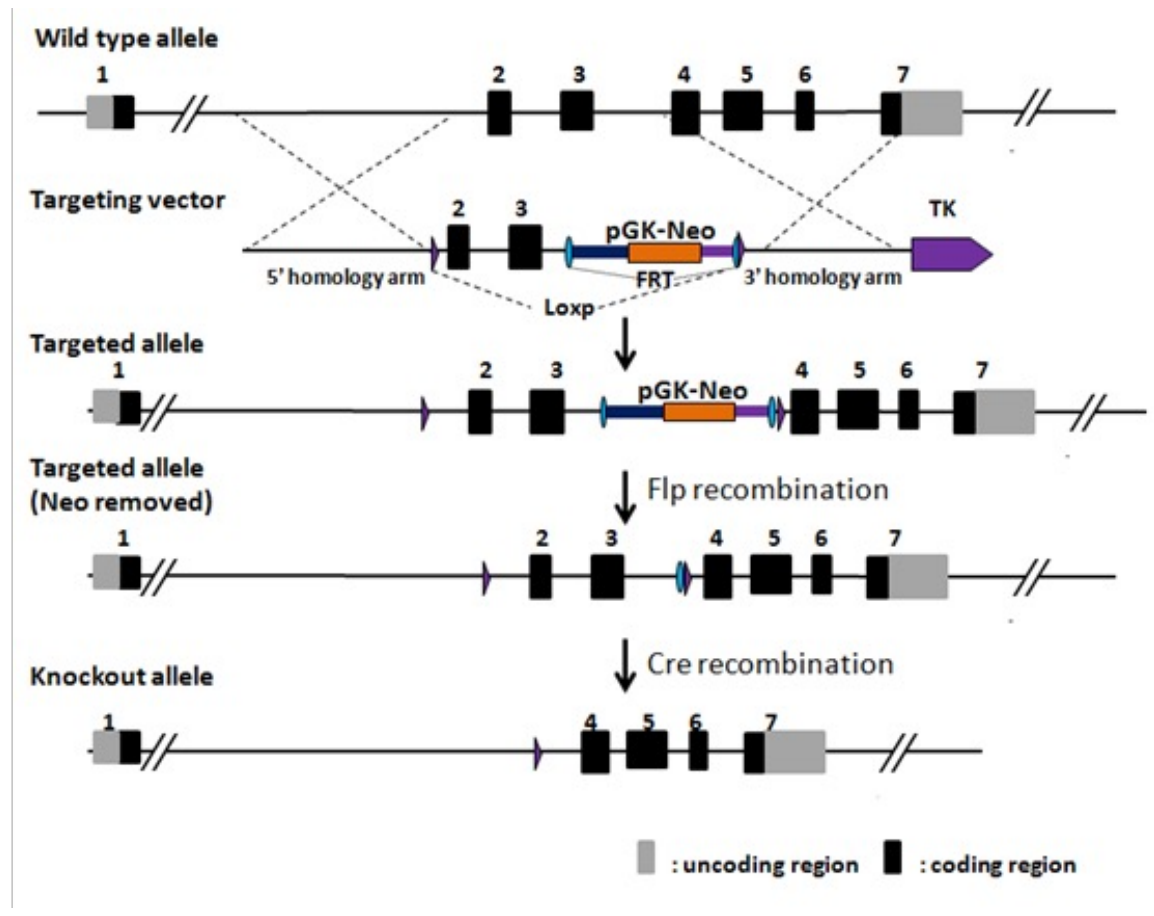

S2B

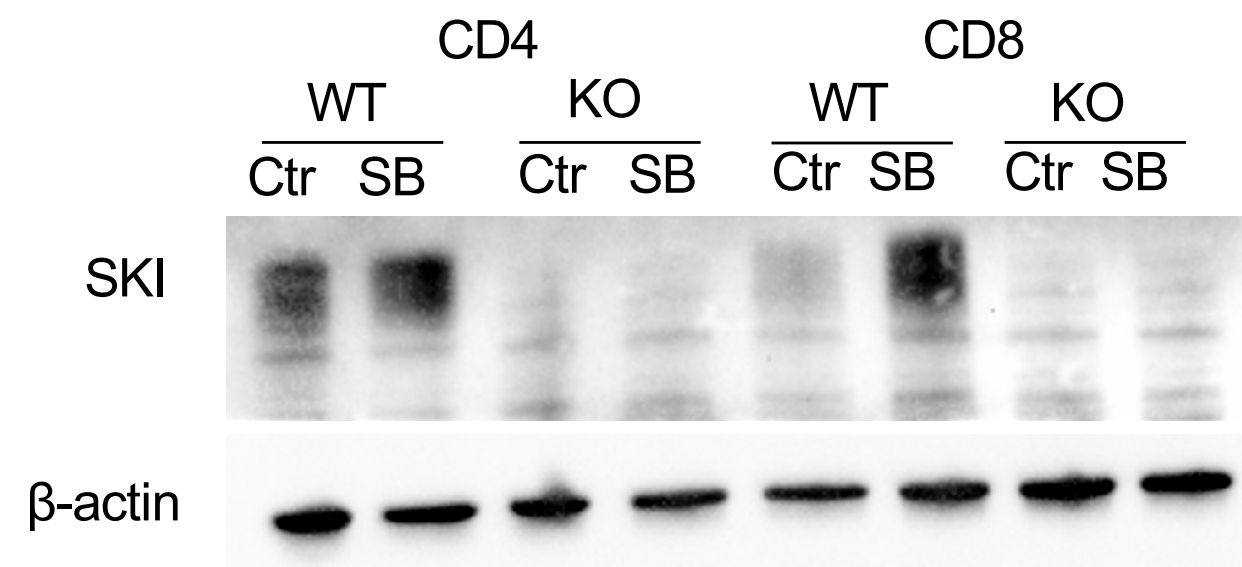

Supplementary Figure S2: Generation of T cell specific SKI knockout mice. (A) Schematic of inserting loxp sites flanking exon 2-3 of endogenous *Ski* allele in mouse genome. **(B)** T cells were recovered from wild-type (wild-type) and *Cd4Cre;Ski*$^{fl/fl}$ (KO) mice. Immunoblotting of SKI protein in CD4+ and CD8+ T cells after being activated with plate-bound anti-CD3 (10 μg/ml) and anti-CD28 (5 μg/ml) for 48 hours in the absence (Ctr) or presence of TGFβR inhibitor SB525334 (10 μM). Results are representative of three experiments.

S3A

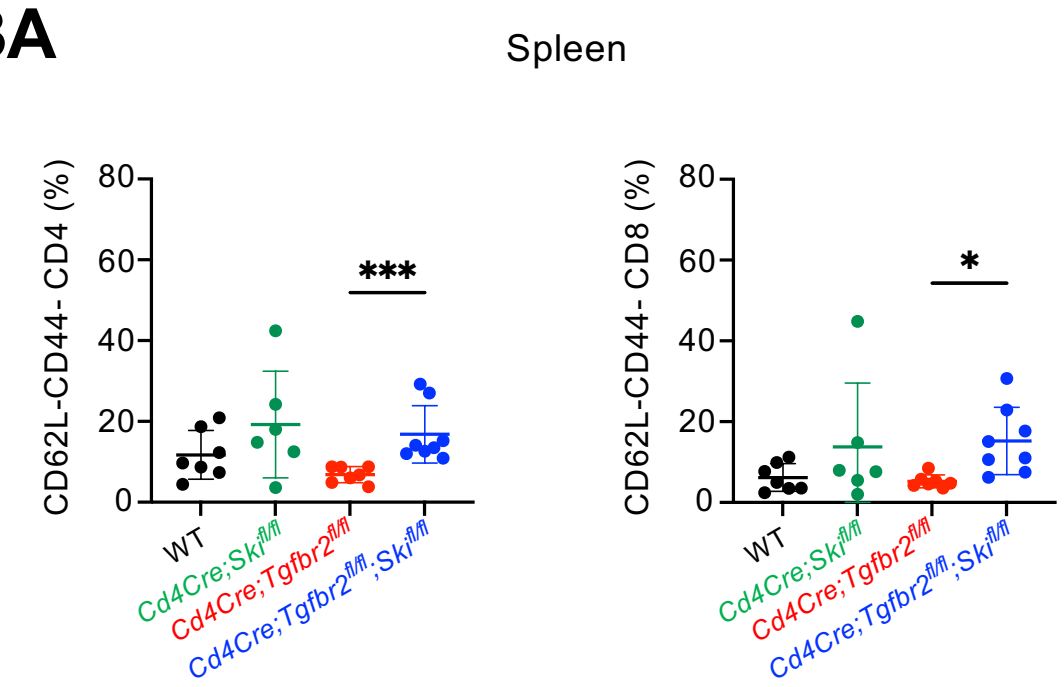

S3B

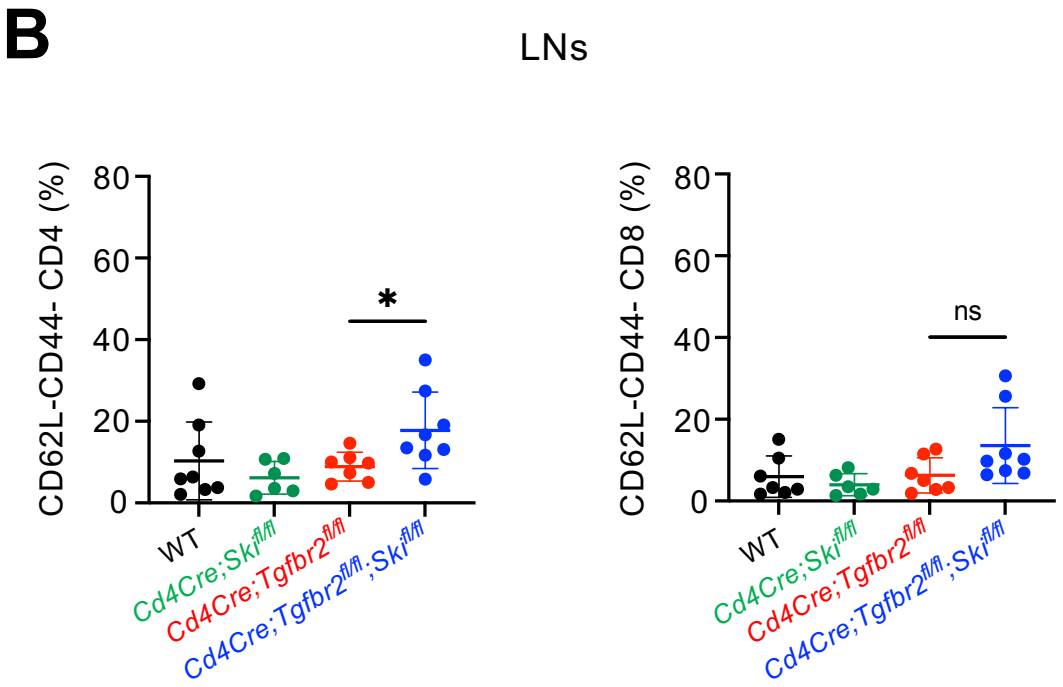

Supplementary Figure S3: CD62L$^-$CD44$^-$ T cell populations in wild-type, *Cd4Cre;Ski$^{fl/fl}$*, *Cd4Cre;Tgfbr2$^{fl/fl}$* and *Cd4Cre;Tgfbr2$^{fl/fl}$;Ski$^{fl/fl}$* mice. A and B, The distribution of CD62L$^-$CD44$^-$ CD4+ and CD8+ T cells in the spleens and peripheral lymph-nodes of wild-type, *Cd4Cre;Ski$^{fl/fl}$*, *Cd4Cre;Tgfbr2$^{fl/fl}$* and *Cd4Cre;Tgfbr2$^{fl/fl}$;Ski$^{fl/fl}$* mice., assayed by flow-cytometry. n>=6 for each group, Means ± SD are shown. *P<0.05; ***P<0.001 per *t* test.

S4A

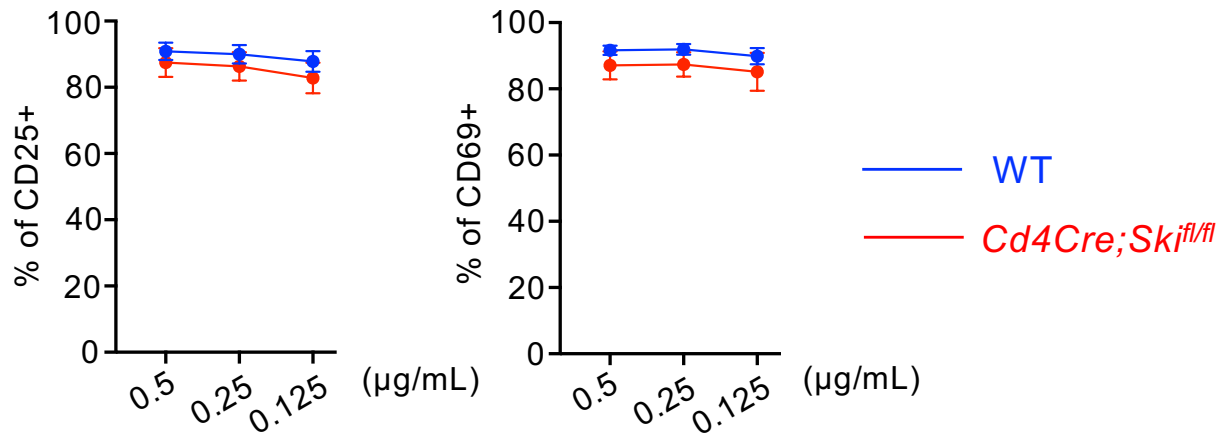

S4B

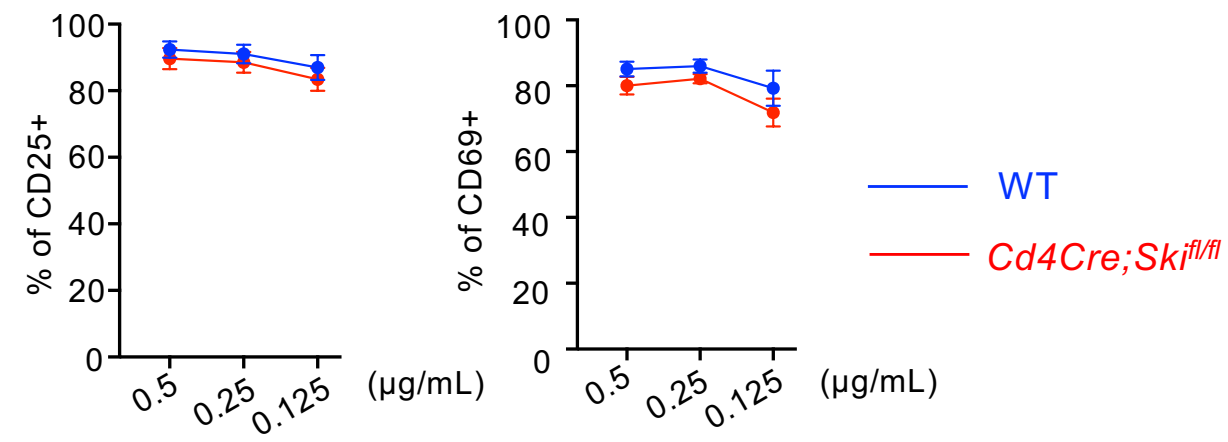

Supplementary Figure S4: The expression of activation markers in SKI-deficient CD4 T cells post activation. CFSE-labeled wild-type (CD45.1, blue lines) and *Cd4Cre;Ski$^{fl/fl}$* (CD45.2, red lines) CD4 T cells were mixed at 1:1 ratio and activated with indicated doses of anti-CD3 in the presence of antigen presenting cells for 12 hours **(A)** and 24 hours **(B)**. The percentages of CD25+ and CD69+ cells were assessed by flow-cytometry. Means ± SD of three repeats. No statistically significant difference was observed.

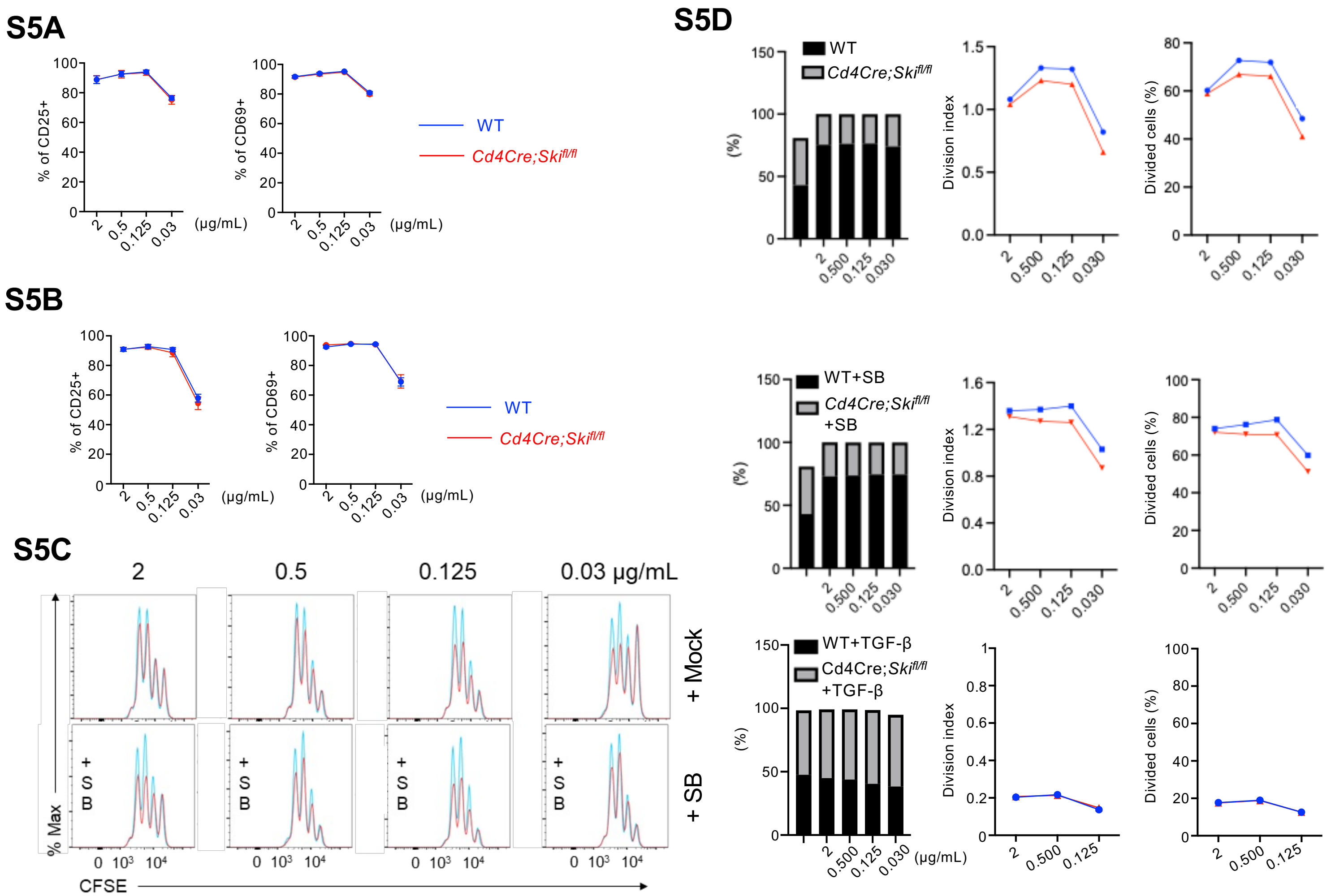

Supplementary Figure S5: CD8 T cell function upon SKI deletion. A and B, CFSE-labeled wild-type (CD45.1, blue lines) and *Cd4Cre;Ski*$^{fl/fl}$ (CD45.2, red lines) CD8 T cells were mixed at 1:1 ratio and activated with indicated doses of anti-CD3 in the presence of antigen presenting cells for 12 hours **(A)** and 24 hours **(B)**. The percentages of CD25+ and CD69+ cells were assessed by flow-cytometry. Means ± SD of three repeats are shown. (**C**) CFSE-labeled wild-type (CD45.1, blue lines) and *Cd4Cre;Ski*$^{fl/fl}$ (CD45.2, red lines) CD8+ T cells were mixed at the ratio or 1:1 and activated for 2 days with indicated doses of anti-CD3 with (+ SB) or without (+ Mock) TGF-β receptor inhibitor SB525334 (10 μM). Cell division was assessed by CFSE dilution and flow-cytometry. Results are representation of three experiments. (**D**) The percentages of co-existing wild-type (CD45.1, blue lines) and *Cd4Cre;Ski*$^{fl/fl}$ (CD45.2, red lines) CD8+ T cells, relative division index, and percentage of divided cells were assessed by flow-cytometry and FlowJo software. Means ± SD of three repeats are shown.

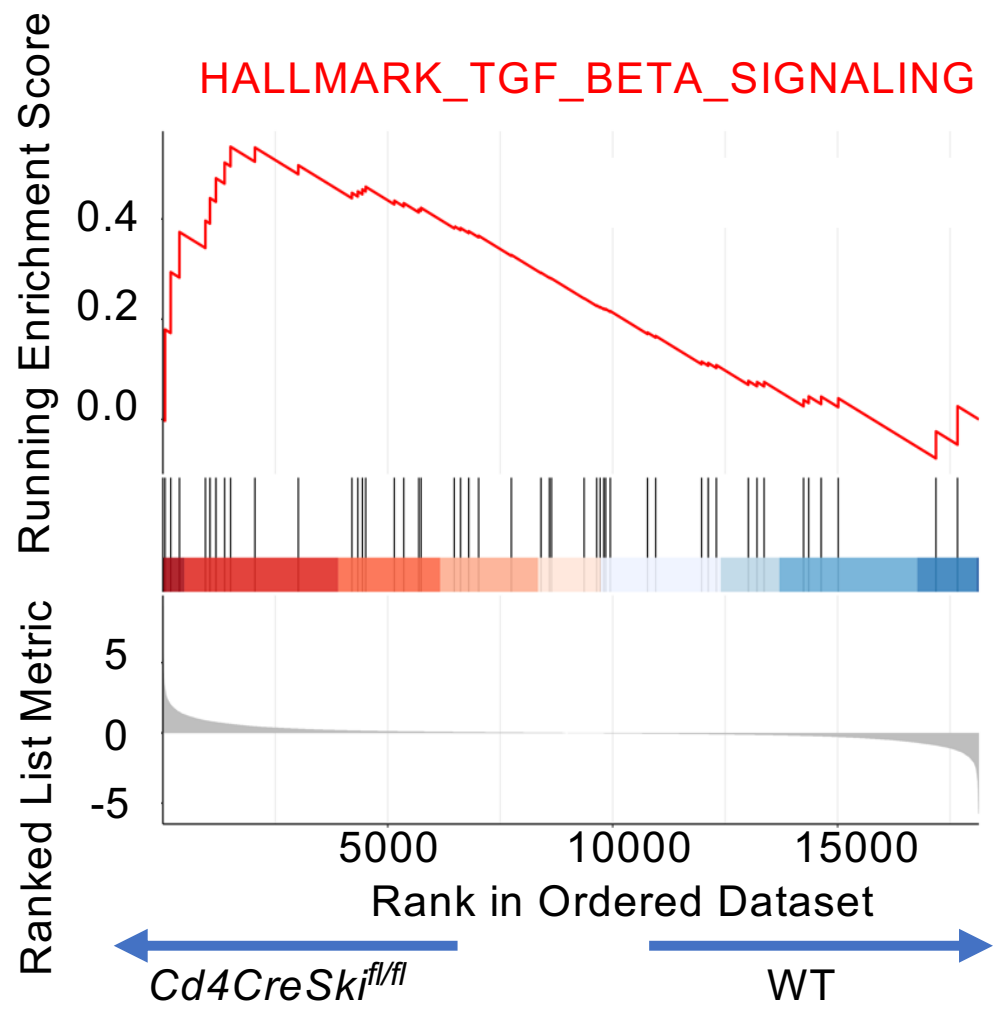

Supplementary Figure S6: The enrichment score of the TGF-β signaling pathway was increased in *Cd4Cre;Ski*$^{fl/fl}$ versus wild-type CD4 T cells after activation. Nominal *P* = 0.03, adjusted *P* = 0.26, NES = 1.50.
